# Antiproliferative and Anti-Migratory Activities of an Extract from *Fridericia platyphylla* Leaves and Its Molecular Profile

**DOI:** 10.3390/plants14172693

**Published:** 2025-08-28

**Authors:** Jhonathas Aparecido R. Brito, Amanda de Jesus A. Miranda, Victor Antonio S. Lima, Samuel dos Santos S. Buna, Marcelino S. do Rosário, Rafael F. Lima, Monique M. Martins, Marcelo S. de Andrade, Maria D. S. B. Nascimento, Vanderlan da Silva Bolzani, Ana Paula Silva de Azevedo-Santos, Josélia Alencar Lima, Júlia Karla de A. M. Xavier, Cláudia Quintino da Rocha

**Affiliations:** 1 Programa de Pós-graduação em Química, Universidade Federal do Maranhão (UFMA), São Luís 65080-805, MA, Brazil; jhonathas.aparecido@discente.ufma.br (J.A.R.B.); miranda.amanda@discente.ufma.br (A.d.J.A.M.); vas.lima@discente.ufma.br (V.A.S.L.); samuel.buna@discente.ufma.br (S.d.S.S.B.); julia.xavier@icen.ufpa.br (J.K.d.A.M.X.); 2 NuBBE, Departamento de Bioquímica e Química Orgânica, Instituto de Química, Universidade Estadual de São Paulo (UNESP), Araraquara 14800-900, SP, Brazil; marcelino.santos@unesp.br (M.S.d.R.); bolzaniv@iq.unesp.br (V.d.S.B.); 3 Laboratório de Cultura de Células (LCC), Programa de Pós-graduação em Saúde do Adulto, Universidade Federal do Maranhão (UFMA), São Luís 65080-805, MA, Brazil; rafaelfrancalima@gmail.com (R.F.L.); mm.martins@discente.ufma.br (M.M.M.); marcelo.andrade@ufma.br (M.S.d.A.); maria.desterro@ufma.br (M.D.S.B.N.); al.joselia@gmail.com (J.A.L.); 4 Departamento de Ciências Fisiológicas, Universidade Federal do Maranhão (UFMA), São Luís 65080-805, MA, Brazil; ana.azevedo@ufma.br; 5 Programa de Pós-graduação em Ciência da Saúde, Universidade Federal do Maranhão (UFMA), São Luís 65080-805, MA, Brazil

**Keywords:** Bignoniaceae, flavonoids, molecular networking, cervical cancer, cell viability

## Abstract

*Fridericia platyphylla*, a member of the Bignoniaceae family, is widely recognized as a rich source of flavonoids with significant biopharmacological potential. This study aimed to perform a chemical annotation of its metabolites and to evaluate the antitumor activity of the hydroalcoholic extract from its leaves. The chemical diversity of this specimen was analyzed using liquid chromatography coupled with tandem mass spectrometry and Molecular Networking. Fifteen significant phenolic compounds were annotated, including phenolic acid derivatives, flavonoid glycosides, and flavone aglycones. Furthermore, the antiproliferative activities against human cervical cell lines, adenocarcinoma HPV 18 positive (HeLa) and carcinoma HPV 16 positive (SiHa), *in vitro*, exhibited distinct inhibitory effects, with IC_50_ values of 44.78 and 66.97 µg mL^−1^, respectively. The extract inhibited cell migration and exhibited cytotoxic effects by reducing the viability of HeLa and SiHa cells, suggesting its potential as a therapeutic candidate for cervical cancer. Therefore, given the significant antiproliferative and anti-migratory activity, these results open up prospects for investigating *F. platyphylla* leaf extract in the development of alternative therapies for cervical cancer.

## 1. Introduction

Cancer is considered a large group of diseases involving abnormal cell growth, which results in an alarming rise in the mortality rate at the worldwide level [1]. Among the types of cancer, cervical cancer is one of the most common malignant forms among women [2]. Due to cervical cancer’s high incidence and mortality, there is an unmet demand for effective diagnostic, therapeutic, and preventive agents [3,4]. Treatments such as chemotherapy, radiation therapy, thermal ablation, and resection have been developed as anticancer therapies. However, toxicities, drug resistance, recurrence, and treatment failure decrease the success rate of these therapies [5,6].

Over the last few decades, the search for natural, plant-derived medicines with anticancer properties has increased as a means of overcoming the adverse side effects associated with cancer therapy. Phytochemicals are promising agents that possess antioxidant, anti-inflammatory, and antiproliferative potential against many cancers, including cervical cancer [4,7]. Studies *in vitro* and *in vivo* demonstrate the chemotherapeutic potential of flavonoids in various types of cancer, including experimental models of lung [8], breast [9], leukemia [10], pancreatic, cervical, ovarian, and prostate cancer [11].

The increased interest in medicinal plants with anticancer properties leads to the exploration of species such as *Fridericia platyphylla* (Cham.) L. G. Lohmann (synonym *Arrabidaea brachypoda*, *A. platyphylla*, *Bignonia brachypoda*, *B. regnelliana*, and *Petastoma simplicifolium*) [12], which stands out in the literature for its therapeutic potential. This species is a vine shrub from the Bignoniaceae family native to the Brazilian savanna “Cerrado” popularly known as “cipó-una”, “tintureiro”, or “cervejinha do campo” [13,14]. A recent study revealed the significant anticancer potential of the flowers, further underlining the importance of *F. platyphylla* in the investigation of natural cancer treatments [15].

Ethanolic extracts from *F. platyphylla* roots exhibit promising cytotoxic and antiproliferative effects. Studies with the crude extract of the roots and the dichloromethane fraction have shown a significant capacity to inhibit the proliferation and migration of gastric, breast, prostate, and cervical tumor cells, reducing cell viability without causing DNA damage [16,17]. In addition, the roots of this plant produce unique glycosylated dimeric flavonoids known as brachydins A, B, and C, which, when isolated, exhibit antitumor properties in prostate cancer cells [18,19].

Despite advances in the phytochemical characterization of *F. platyphylla* roots and flowers, the chemical profile of the leaves remains poorly investigated in the context of their anticancer biological activities. Most studies focus on other parts of the plant, particularly the presence of compounds such as brachydins, whose bioactive activity against tumor cells has already been demonstrated [18,19]. However, the evaluation of these effects in the leaf extract remains underexplored, making this work innovative in exploring the potential of this extract for the treatment of cervical cancer. Considering the importance of the leaves as an abundant source of flavonoids, the present study seeks to fill this knowledge gap by analyzing the phytochemical composition of the leaf extract and evaluating its antiproliferative and anti-migratory effects *in vitro* in human cervical cancer cell lines HeLa and SiHa.

## 2. Results and Discussion

### 2.1. Chemical Profile of Crude Extract of Fridericia Platyphylla Leaves (FAB)

**Figure 1:**
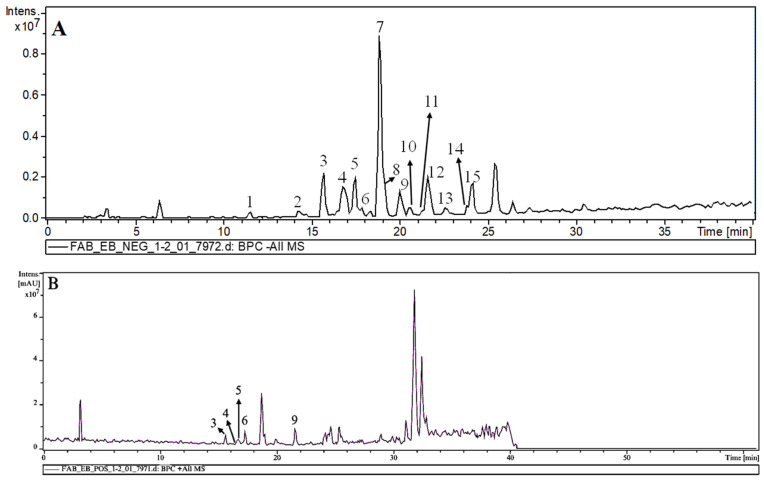
Base peak chromatogram (**A**) (negative mode), (**B**) positive mode of the extract from *Fridericia platyphylla* leaves. The peak numbers in this figure correspond to those used in Table 1.

**Figure 2:**
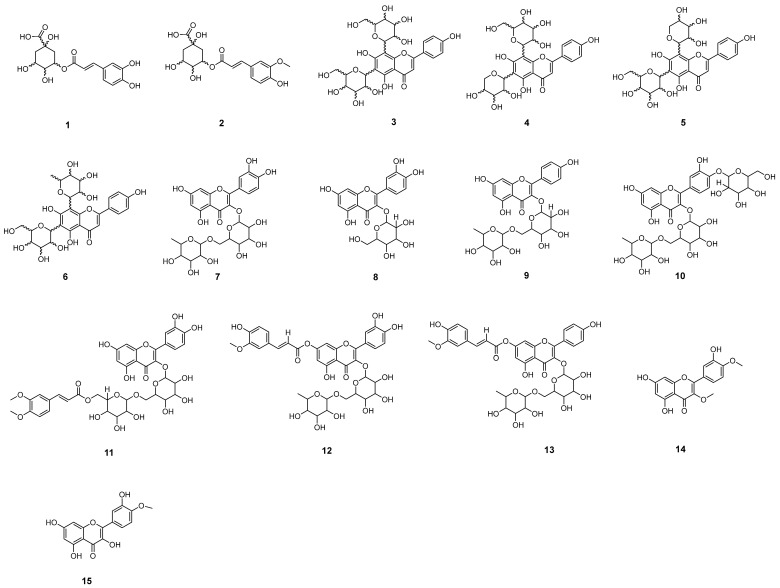
Chemical constituents annotated in the hydroethanolic extract of *F. platyphylla* leaves: (**1**) caffeoylquinic acid; (**2**) feruloylquinic acid; (**3**) apigenin 6,8-*C*-dihexoside; (**4**) apigenin 6-*C*-pentosyl-8-*C*-hexoside; (**5**) apigenin 6-*C*-hexosyl-8-*C*-pentoside; (**6**) apigenin 6-*C*-hexosyl-8-*C*-deoxihexoside; (**7**) rutin; (**8**) quercetin-*O*-hexoside; (**9**) kaempferol-*O*-deoxyhexosyl-*O*-hexoside; (**10**) quercetin -*O*-deoxyhexosyl-*O*-dihexoside; (**11**) quercetin-*O*-dimethoxycaffeoyl-*O*-hexosyl-*O*-hexoside; (**12**) quercetin-*O*-feruloyl-*O*-deoxyhexosyl-*O*-hexoside; (**13**) kaempferol-*O*-feruloyl-*O*-deoxyhexosyl-*O*-hexoside; (**14**) 3,4′-dimethoxy-quercetin; (**15**) tamarixetin.

MS/MS analyses of FAB were conducted in both negative and positive modes. Compound structures were proposed based on fragments derived from protonated/desprotonated molecules via liquid chromatography coupled to mass spectrometry (HPLC-MS/MS, Figure 1), as evidenced in Table 1, revealing the presence of fifteen phenolic compounds, including chlorogenic acids and glycosylated flavonoids belonging to the subclass of flavones and flavonols. Figure 2 illustrates the proposed molecules established through a comparison of the data with fragmentation profiles available in the literature.

### 2.2. Chromatographic Peaks Annotation

#### 2.2.1. Chlorogenic Acids

Compound (**1**), identified as caffeoylquinic acid, was initially observed with a deprotonated precursor ion peak [M − H]^−^ at *m*/*z* 355; however, after inspection of the spectrum, this value was corrected to *m*/*z* 353, as the discrepancy was caused by a calibration offset in the instrument. The compound exhibited a retention time (Rt) of 11.6 min and was annotated in the FAB extract (see Figure S1 and Scheme S1, Supplementary Information). The ion at *m*/*z* 191 [M − H − 162]^−^ indicates the loss of 162 Da, corresponding to the caffeoyl portion. This loss is easily observed due to the capture of hydrogen from the phenolic hydroxyl by oxygen bonded to quinic acid [35]. Additionally, the ion at *m*/*z* 179 [M − H − 174]^−^ represents the loss of the quinic acid portion, indicating cleavage of the C-O bond, which has lower stability in ion formation.

Compound (**2**), annotated in negative mode, displays a mass spectrum with a deprotonated precursor ion initially observed at *m*/*z* 369; however, this value should correspond to *m*/*z* 367, as the instrument was not calibrated and presented a +2 Da error. This ion is characteristic of one of the isomers of feruloylquinic acid at a retention time of 14.2 min (see Figure S2 and Scheme S2, Supplementary Information). This molecule is derived from cinnamic acid, as confirmed by the MS^2^ stage, which revealed a fragment at *m*/*z* 193 (base peak). This fragment corresponds to the loss of 174 Da [M − H − 174]^−^, indicating the cleavage of the ester bond between the dehydrated quinic acid and the feruloyl substituent. In the MS^3^ stage, the first fragmentation resembles that observed in the MS^2^ stage, producing a fragment of 174 Da. The precursor ion at *m*/*z* 149 [M − H − 44]^−^ was generated, resulting from the cleavage of the carboxylic group in the feruloyl acid molecule. Subsequently, a methyl group was lost, resulting in a precursor ion at *m*/*z* 134. Finally, a loss of 17 Da occurred, corresponding to a radical loss of the hydroxyl.

#### 2.2.2. Glycosylated Flavones

Chemical characterization of glycosylated flavonoids in mixtures poses a challenge due to the degree of isomerization in the aglycone and the sugar units detected within the molecule [36]. During the cleavage of *O*-glycosidic bonds between flavonoids and sugars, it is observed that the number of sugar moieties attached to an aglycone can vary from one to five units, allowing for the identification of cleavages resulting in losses of hexosides (162 Da), deoxyhexosides (142 Da), and pentosides (132 Da) [37]. *C*-glycosylated flavonoids, which feature C-C bonds exclusively occurring at positions C6 and/or C8, exhibit a characteristic fragmentation pattern. The fragmentation of these compounds reveals distinctive ions at [M − H − 210]^−^, [M − H − 90]^−^, and [M − H − 60]^−^, as observed in previous studies [24].

Four compounds derived from apigenin were observed: apigenin 6,8-*C*-dihexoside (**3**), apigenin 6-C-pentosyl-8-*C*-hexoside (**4**), apigenin 6-*C*-hexosyl-8-*C*-pentoside (**5**), and apigenin 6-*C*-hexosyl-8-*C*-deoxyhexoside (**6**). Compound (**3**), when ionized in negative (see Figure S3 and Scheme S3, Supplementary Information), was annotated as apigenin 6,8-*C*-dihexoside [24]. This exhibited a precursor ion [M − H]^−^ with *m*/*z* 593 and displayed a typical fragmentation pattern in the MS^2^ stage of *C*-di-glycosides. Such fragmentation included losses of single sugar *C*-glycosidic bonds, such as cleavage at the 0.2 × 6 bond [M − H − 120]^−^. In positive mode (see Figure S16 and Scheme S16, Supplementary Information), a protonated precursor ion was observed at *m*/*z* 595. The initial fragments were characterized by successive dehydrations [M + H − 18 − 18]^+^, resulting in ions at *m*/*z* 577 and *m*/*z* 559, respectively. The precursor ion peak at *m*/*z* 595 underwent a loss of 120 Da from the *C*-glycosidic bond of the hexose. This 120 Da loss similarly occurred from the ion at *m*/*z* 577, yielding a fragment at *m*/*z* 457.

Compound (**4**), ionized in both negative (see Figure S4 and Scheme S4, Supplementary Information) and positive modes, was annotated as apigenin 6-*C*-pentosyl-8-*C*-hexoside with a retention time of 16.7 min. In negative mode, the precursor ion *m*/*z* 563 resulted in ions *m*/*z* 545, *m*/*z* 473, and *m*/*z* 443, corresponding to losses of 18 Da, 90 Da, and 120 Da, respectively. From *m*/*z* 473, through homolytic cleavages and hydrogen rearrangements, there was a neutral loss of 88 Da from the sugar unit, yielding a precursor ion with *m*/*z* 385. Subsequently, a loss of 30 Da, corresponding to formaldehyde (CH_2_O), generated *m*/*z* 355. In positive mode (see Figure S17 and Scheme S17, Supplementary Information), the protonated precursor ion *m*/*z* 565 underwent successive dehydrations [M + H − 18 − 18]^+^, producing ions *m*/*z* 547 and *m*/*z* 529. From *m*/*z* 529, a loss of 100 Da occurred due to hydrogen rearrangement in the sugar unit, resulting in the ion *m*/*z* 429, which then underwent dehydration, generating the ion *m*/*z* 411.

Compound (**5**) (Rt = 16.9 min), annotated as apigenin 6-*C*-hexosyl-8-*C*-pentoside, displayed fragments similar to those observed for molecule **4** (see Figure S5, Scheme S5, Figure S18, and Scheme S18, Supplementary Information). Compound (**6**), ionized in both negative and positive modes with a Rt of 17.4 min, was identified as apigenin 6-*C*-hexosyl-8-*C*-deoxyhexoside. In negative mode (see Figure S6 and Scheme S6, Supplementary Information), starting from the precursor ion *m*/*z* 577, two fragmentation pathways were proposed. In the first pathway, a loss of 90 Da occurred, generating the ion *m*/*z* 487, while in the second pathway, a loss of 120 Da resulted in the most abundant peak, *m*/*z* 457. In positive mode (see Figure S19 and Scheme S19, Supplementary Information), with the protonated precursor ion *m*/*z* 579, the fragmentation patterns were similar to those observed for compounds **4** and **5** [M + H −18 − 18 −100]^+^, yielding the ions *m*/*z* 561, *m*/*z* 543, and *m*/*z* 443.

#### 2.2.3. Glycosylated Flavonols

The compound rutin (**7**) (Rt = 18.9 min) displayed a precursor ion [M − H]^−^ with *m*/*z* 609, while the MS^2^ ion, initially observed at *m*/*z* 303, corresponds in fact to *m*/*z* 301 due to a calibration offset in the instrument. This ion was attributed to [M − H − 308]^−^, representing the loss of 308 Da corresponding to the rhamnose (146 Da) plus glucose (162 Da) moieties [35]. This fragmentation is characteristic and contributes to the annotation of rutin and the understanding of its structure [27] (see Figure S7 and Scheme S7, Supplementary Information). It is a phytochemical of specific interest due to its wide range of beneficial pharmacological activities. These properties encompass anticancer, antimicrobial, anti-inflammatory, antioxidant, cardiovascular, and antidiabetic activities, rendering it a highly significant flavonoid in the pharmaceutical industry [38].

Quercetin-*O*-hexoside (**8**) (Rt = 19.1 min) revealed that the deprotonated molecule exhibited the precursor ion [M − H]^−^ at *m*/*z* 463. In this deprotonation process, two hydrogens belonged to the glycoside and one to the aglycone. Upon loss of the hexose fragment [M − H − 162]^−^, which corresponds to cleavage at the C-O linkage at the hydroxyl group of C3 in the C ring of the aglycone [30], the resulting ion should be *m*/*z* 301; however, due to instrument miscalibration, it was observed at *m*/*z* 303, reflecting a +2 Da error. This fragmentation pattern provides structural information and the location of the bond in the quercetin-*O*-hexoside molecule (see Figure S8 and Scheme S8 in the Supplementary Information).

Compound (**9**) (Rt = 20.0 min) was ionized in negative and positive modes. In the MS^2^ stage (see Figure S9 and Scheme S9, Supplementary Information), with the deprotonated precursor ion peak at *m*/*z* 593, the presence of two sugar residues (pentose and hexose) linked to the aglycone on the same carbon was observed. Upon loss of 308 Da, corresponding to these sugar residues, the resulting ion should be *m*/*z* 285; however, due to instrument miscalibration, it was observed at *m*/*z* 287, reflecting a +2 Da error. This fragment was annotated as kaempferol-*O*-deoxyhexosyl-*O*-hexoside [32]. On the other hand, in the MS^2^ stage, from the protonated precursor ion with *m*/*z* 595 it was possible to propose three fragmentation pathways, [M + H − 46]^+^, [M + H − 146]^+^ and [M + H − 308]^+^, recording the ions *m*/*z* 549, 449 and 287, respectively (see Scheme S20, Supplementary Information).

Compound (**10**) (Rt = 20.5 min) exhibited a deprotonated precursor ion [M − H]^−^ with *m*/*z* 771, involving the loss of two hydrogens from the glycoside and one from the aglycone. In the MS^2^ stage, the presence of a fragment ion at *m*/*z* 609 [M − H − 162]^−^ was observed, indicating the removal of a glycosidic portion, more precisely a hexose linked to the flavonoid part of the molecule. From the precursor ion at *m*/*z* 609, cleavage occurred at the C-7 position of the C-O linkage, producing a fragment that should correspond to *m*/*z* 301 upon loss of the glucose at C3; however, due to instrument miscalibration, it was observed at *m*/*z* 303, reflecting a +2 Da error. Based on this data, the presence of the molecule quercetin-*O*-deoxyhexosyl-*O*-dihexoside is suggested, as indicated by the MS/MS analysis (see Scheme S10, Supplementary Information).

Compound (**11**) (Rt = 21.3 min) was proposed as quercetin-*O*-dimethoxycaffeoyl-O-hexosyl-O-hexoside, since it generated a deprotonated precursor ion peak [M − H]^−^ initially observed at *m*/*z* 817; however, this value should correspond to *m*/*z* 815 due to instrument miscalibration, reflecting a +2 Da error. In the MS^2^ stage, fragments at *m*/*z* 206 [M − H − 206]^−^ were observed, corresponding to the loss of a dimethoxycinnamoyl portion, and at *m*/*z* 609 [M − H − 18]^−^, indicating the elimination of a water molecule. Additionally, a precursor ion at *m*/*z* 303 [M − H − 162 − 146]^−^ was recorded in the MS^3^ stage; this ion should correspond to *m*/*z* 301, but due to the same calibration offset, it was observed at *m*/*z* 303. These losses involved the dimethoxycinnamoyl group and two sugar units linked to each other, comprising hexose (162 Da) and rhamnose (146 Da) (see Figure S11 and Scheme S11, Supplementary Information).

Through a detailed analysis of the MS^2^ stage, it was possible to annotate compounds such as quercetin-*O*-feruloyl-*O*-deoxyhexose-*O*-hexoside (**12**) (Rt = 21.5 min). This annotation was based on the observation of a retention peak at 21.6 min, along with a deprotonated precursor ion peak [M − H]^−^ *m*/*z* 785. During the analysis, the occurrence of an ion fragment at *m*/*z* 609 was noted after the feruloyl fraction fragmentation, representing a loss of 176 Da [M − 176]^−^. Additionally, a precursor ion at *m*/*z* 303 was recorded, corresponding to a loss of 306 Da [M − H − 162 − 146]^−^, indicating the presence of hexose (162 Da) and deoxyhexose (146 Da) in the compound’s composition. This ion should be *m*/*z* 301 upon loss of the glucose at C3; however, due to instrument miscalibration, it was observed at *m*/*z* 303, reflecting a +2 Da error (see Figure S12 and Scheme S12, Supplementary Information).

Peak (**13**), presented at a Rt of 22.6 min, revealed a precursor ion with *m*/*z* 769 [M − 2H]^−^. The loss of a feruloyl portion was identified, resulting in [M − H − 176]^−^ and in the registration of the precursor ion *m*/*z* 593. Furthermore, the base peak at *m*/*z* 287 was observed, corresponding to a loss of 306 Da [M − H − 162 − 146]^−^, representing the elimination of a hexose (162 Da) and a deoxyhexose (146 Da). This ion should be *m*/*z* 285 upon cleavage at C3; however, due to instrument miscalibration, it was observed at *m*/*z* 287, reflecting a +2 Da error. It was possible to annotate compounds such as quercetin-*O*-kaempferol-*O*-feruloyl-*O*-deoxyhexosyl-*O*-hexoside (see Figure S13 and Scheme S13, Supplementary Information).

#### 2.2.4. Flavonol Aglycone

In the mass spectrum of compound (**14**) (Rt = 23.8), conducted in negative ionization mode (see Scheme S14, Supplementary Information) [M − H]^−^, the presence of the base peak ion at *m*/*z* 331 was evident, along with its fragment at *m*/*z* 314, generated by the loss of a methyl group, resulting in [M − H − 15]^−^. This ion should actually be *m*/*z* 329, but due to instrument miscalibration, it was observed at *m*/*z* 331, reflecting a +2 Da error. However, only the base peak ion at *m*/*z* 331 was present in positive ionization mode.

Compound (**15**) (Rt = 25.5) was proposed as tamarixetin, with a [M − H]^−^ ion at *m*/*z* 315, which appeared as *m*/*z* 313 due to a +2 Da error caused by instrument miscalibration. It presented a fragment ion at *m*/*z* 300, indicating a loss of 15 Da due to cleavage at the C8 of the C–O linkage (see Scheme S15, Supplementary Information).

### 2.3. Global Natural Products Social Molecular Networking (GNPS)

**Figure 3:**
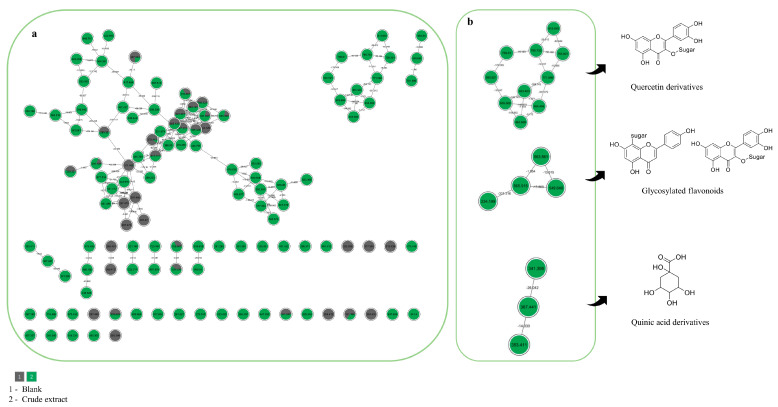
(**a**) Complete molecular network obtained; (**b**) focus on molecular families obtained from classical molecular networking and annotated based on spectral matches within the GNPS platform. Each node represents a cluster of tandem mass spectrometry spectra (MS/MS), while the edges that connect them represent the MS/MS fragmentation similarity (cosine > 0.7).

Besides the analysis of the raw data (MS^2^ and MS^3^), an untargeted analysis was performed using the mzML files on the GNPS platform. This approach was employed to support the chemical characterization and complement the diversity of secondary metabolites, and the results were compared to MS annotations. In this context, a small number of library matches, using the previous chemical analysis, a manual inspection in each node and spectral family (Figure 3) was made to verify and confirm the compounds previously annotated in raw data (Table S1, Supplementary Information). The use of the GNPS platform allows the expansion of the annotations based on raw data and organizes the data more efficiently, based on *m*/*z* values and fragmentations.

The GNPS public spectral reference libraries were searched using the MS/MS spectra, and molecular networks were built to visualize the results. Other compounds were also annotated via GNPS platform, such as tricin-*C*-hexoside, apigenin-*C*-hexoside, kaempferol-*O*-hexoside, and some taxifolin derivatives, and some of the previously annotated compounds were confirmed by molecular networking, as shown in Table S1 (Supplementary Information). The molecular networking analysis allowed the annotation of 15 compounds, including a chemical diversity of flavonoid structures, based on their similar pattern of fragmentation, and some related in the literature. Figure 3 presents the molecular networks of spectral families generated after manually evaluating the annotated compounds through mass spectra. It is possible to verify and confirm that the FAB is rich in phenolic compounds, especially flavonoids and phenolic acids. However, the molecular network analysis was used as a complementary technique to the chemical inspection of raw data.

These results reinforce the chemical complexity of the analyzed extracts and demonstrate the utility of molecular networking as a strategy to explore minor compounds or those not initially detected through conventional inspection. Furthermore, the confirmation of key phenolic markers through GNPS supports the robustness of the raw data annotation and enhances confidence in the chemical profiles generated. This integrative approach thus provides a broader and more accurate understanding of metabolite diversity, which is essential for future pharmacological or ecological studies involving these species.

The most common polyphenols in plants are stilbenes, flavonoids, and phenolic acids. Approximately 30% to 60% correspond to phenolic acids and flavonoids, respectively [39]. In general, the presence of *C*-glycosylated flavonoids is reported in *Arrabidaea samydoides*, *Arrabidaea chica,* and *Arrabidaea patellifera* [14]. These flavonoids can be considered chemical markers, primarily due to their chemical stability and structural diversity [40]. The flavonoids, such as apigenin and rutin, are commonly identified in *F. platyphylla* leaf extracts [14].

The chemical analysis of the *F. platyphylla* extract performed in this study revealed a phytochemical profile consistent with data previously described in the literature. Flavonoids such as apigenin and rutin are compounds commonly reported in leaf extracts of this species [14]. Moreover, previous studies aimed to isolate and characterize rare dimeric flavonoids, known as brachydins [41], which have relevant biological activities, highlighting their anticancer, antioxidant, and anti-inflammatory effects [15]. In the present work, in addition to the identification of these previously described compounds, less common glycosylated flavonoids were also provisionally noted, including apigenin 6,8-*C*-dihexoside, apigenin 6-*C*-pentosyl-8-*C*-hexoside, as well as acylated derivatives of quercetin and kaempferol, such as quercetin-*O*-feruloyl-*O*-deoxyhexosyl-*O*-hexoside, thus expanding the known phytochemical profile for the species.

### 2.4. Cytotoxic Effect of FAB on HeLa and SiHa Cell Lines

**Figure 4:**
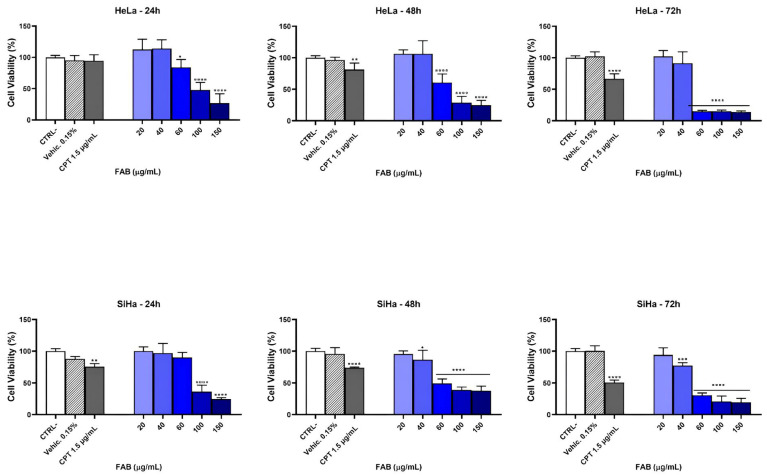
Analysis of cell viability and IC_50_ of *F. platyphylla* in SiHa and HeLa cells: cytotoxicity of HeLa cell line; cytotoxicity of SiHa cell line. FAB: crude ethanolic extract; CTRL^−^: control negative; CPT (Cisplatin); SiHa: carcinoma; HPV 16 positive; HeLa: adenocarcinoma HPV 18 positive. The frequency analysis between the groups was compared using the ANOVA test with multiple comparisons, considering *p*-values * *p* < 0.05, ** *p* < 0.001, *** *p* < 0.0009, and **** *p* < 0.0001.

The antiproliferative activity of FAB in HeLa and SiHa cells is shown in Figure (Figure 4). Cell viability was inhibited at the highest concentrations of FAB (150 µg mL^−1^). HeLa cells treated with FAB at 24, 48, and 72 h presented an inhibitory concentration, respectively, 78.78 µg mL^−1^, 59.52 µg mL^−1^, and 44.78 µg mL^−1^, compared to SiHa cells treated with FAB, the inhibitory concentrations at 24, 48, and 72 h were 81.90 µg mL^−1^, 50.01 µg mL^−1^, and 66.97 µg mL^−1^. The results presented reduced viability in a concentration- and time-dependent manner, presenting values < 100 µg mL^−1^, appearing as active [42]. As shown in Figure 4, the extract reduced viability in a concentration and time-dependent manner. Even though the IC_50_ value was lower in HeLa, suggesting greater sensitivity of this cell line compared to SiHa, there was no significant difference (Table 2).

The cytotoxicity experiment was carried out on HeLa and SiHa cell lines, which are HPV+ (Human Papillomavirus positive) [43]. The results showed that the extract reduced cell viability in both cell lineages and with IC_50_ values that were close to each other. HPV infection is considered one of the main factors associated with pre-neoplastic changes in cervical cancer [44]. Viral insertion induces the production of proteins, mainly E6 and E7, which increase the replicative capacity and immortalization of the cell [45].

The results show the promising effect of the FAB on two types of lineages representing different histological origins, carcinoma (SiHa) and adenocarcinoma (HeLa), and can be applied to various types of cervical cancer. On the other hand, the data suggests that a possible target of the compounds present in the extract may be related to the presence of HPV. Several studies have shown the effectiveness of natural products in HPV^+^ tumors, such as flavonoids, tannins, alkaloids, and phenolic compounds, among others, with the mechanism of viral infection as an antineoplastic action [46]. We therefore assume that the compounds present in FAB are capable of inducing cytotoxicity through mechanisms associated with HPV infection.

Considering its natural source alongside minimal toxicity level, the chlorogenic acids (caffeoylquinic and feruloylquinic acids), two of the main groups found in green coffee, have gained considerable attention nowadays from researchers worldwide, owing to their wide, efficacious, and beneficial action against cancer. The application these compounds in cancer treatment has been enormously reported and demonstrated in several cell lines of cancer, such as colon (MCF-7, HT-29, CT-26, and HCT-116), liver (HepG2), blood (U937, HL-60, Bcr-Abl, and K-562), bone (U2OS, Saos-2, and MG-63 OS), pancreatic (PANC-1), and skin (SK-MEL-2) [47].

Another class that attracts the attention of researchers due to its wide range of beneficial effects on human health and low toxicity is the flavonoids. In addition to the main subgroup of flavonoids, their glycosides also receive increasing attention. These compounds possess similar stability and bioactivity but usually have improved solubility, reduced biological toxicities, and are often more efficacious than their non-glycoside forms in pharmacological studies. Tests performed on RAW 264.7 murine macrophage cells showed that flavonoid glycosides exhibited more significant anti-inflammatory activities compared to their related aglycones [48].

In the literature, rutin identified in *F. platyphylla* extract is described as a glycosidic flavonol belonging to an important class of flavonoids. This compound stands out for its therapeutic relevance, acting to improve capillary resistance and permeability, in addition to exhibiting antioxidant, anti-inflammatory, and anticarcinogenic activities. It also demonstrates antimicrobial activity against *Pseudomonas aeruginosa* and *Staphylococcus aureus* [49].

Similarly, Brachydin A (BrA), glycosylated flavonoids of a dimeric nature, also found in this plant species, have shown promising antitumor activity. *In vitro* studies indicate that these substances induce cytotoxicity and cell death by apoptosis and necrosis in metastatic prostate cells (PC3), in addition to reducing cell migration. They exhibit lower toxicity to non-tumor prostate cells (PNT2), suggesting selectivity for mPCa tumor cells [50].

### 2.5. Wound Healing Model

**Figure 5:**
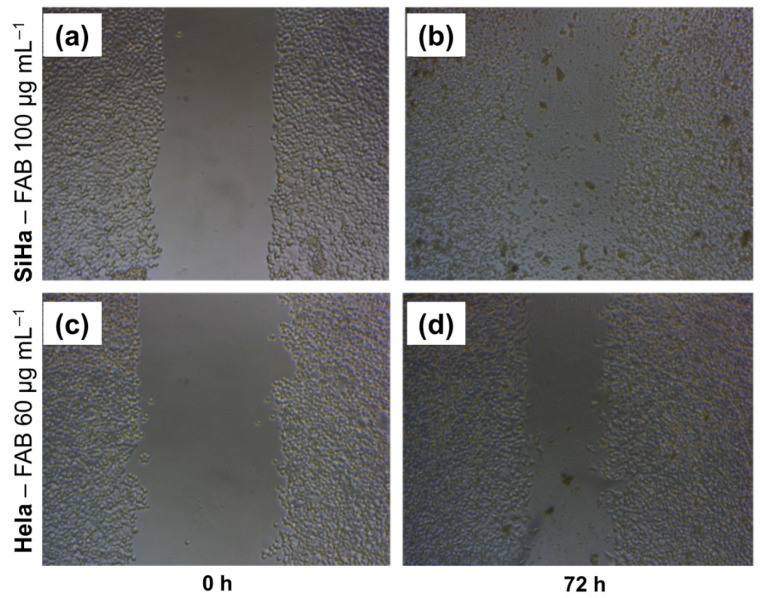
Effect of *F. platyphylla* on migration of SiHa and HeLa cells: (**a,b**) SiHa treated with *F. platyphylla* at 0 h and 72 h, respectively; (**c**,**d**) HeLa treated with *F. platyphylla* at 0 h and 72 h, respectively.

The MTT assay measures cell viability through metabolic activity. To further investigate the extract’s effect, a wound healing/migration protocol was performed to assess the proliferation and migration capacity of tumor cells. It was observed that the HeLa cell line was more sensitive to FAB treatment than the SiHa cell line for a time range of 0 to 72 h. Since the HeLa cell line has a greater migratory effect than SiHa, we can conclude that FAB treatment is effective in inhibiting the migratory capacity of cell lines with a high migratory and proliferative effect (Figure 5). For the cytotoxic effect, the cell lines were treated with the extract at a concentration of 20–100 µg mL^−1^. In the HeLa cell line, treatment with the extract at a concentration of 100 µg mL^−1^ showed a reduction in proliferation at all time points, and at a concentration of 60 µg mL^−1^, it showed an antiproliferative effect at 48 and 72 h, depending on the time and concentration. The SiHa cell line proved to be more sensitive, with a reduction in proliferation at concentrations of 60 and 100 µg mL^−1^ in 24 h and at a concentration of 40 µg mL^−1^ in 48 and 72 h, depending on the time and concentration (Table 3).

Collective cell migration is a key feature of cancer invasion and metastasis, involving dynamic interactions and cross-interactions between cells and the extracellular matrix, as well as the regulated production of soluble mediators and cytokines [51,52]. Thus, the analysis of cell migration *in vitro* is a useful assay for quantifying changes in the migratory capacity, and the “*in vitro* scraping assay” is a simple, versatile, and economical method [51]. At the two highest concentrations, the extract showed a static effect on the cells’ ability to migrate and close the “wound”. The presence of flavonoids in extracts obtained from plants has been shown to exhibit various activities, including reducing the migratory capacity and, consequently, metastasis in tumors associated with HPV infection [53,54].

The results showed that the extract had anti-migratory potential in both cervical cancer cell lines. However, the SiHa showed more interesting results compared to HeLa at the same concentrations. In cervical cancer, the presence of E6/E7 viral proteins is associated with greater expression of chemokine receptors that regulate different intracellular signaling pathways associated with proliferation, tumor aggressiveness, and metastasis [55]. On the other hand, the treatment was more effective in SiHa than in HeLa cells, which may be associated with the type of HPV each cell line has. Similar results were also observed with quercetin, which was more active in SiHa than in HeLa in terms of inducing effects such as cell cycle arrest and apoptosis [56].

Cell migration and proliferation are cellular processes induced by the relief of contact inhibition and are confounding factors in wound healing trials [51]. Thus, the antiproliferative effect observed in the MTT assay may corroborate the reduction in wound closure, added to the anti-migratory effect. Previous studies have shown that the action of chemical compounds and natural products on HPV-infected cell lines (HeLa, SiHa and Caski) showed: a reduction in the mRNA levels of E6 and E7, inhibition of the function of E6/E7 in binding to p53, restoration of p53 expression, inhibition of proliferation of treated cells, induction of apoptosis [45]. The data suggest that the compounds in the extract could have the capacity to inactivate viral mechanisms.

The literature proves the action of quercetin, taxifolin, and rutin acting on several fronts to combat HPV-infected cells and promote beneficial effects, such as the restoration of p53 expression and the induction of apoptosis [56,57,58]. In general, natural bioactive compounds, especially the flavonoids, have a significant impact on the treatment of carcinoma. They exhibit strong antioxidant activity and neutralize the effects of free radicals due to the presence of hydroxyl groups, and they chelate metal ions [59]. Taxifolin has shown promising inhibitory activity against inflammation, malignancies, microbial infection, oxidative stress, cardiovascular disease, and liver disease. Anticancer activity is relatively significant than other activities investigated *in vitro* and *in vivo* with little or no side effects to the normal healthy cells [60]. Ovarian cancer cells are willing to establish resistance to common cancer therapies. Several *in vitro* and *in vivo* studies were conducted to evaluate cytotoxic effects of quercetin on ovarian cancer. Since quercetin does not harm healthy cells and it is cytotoxic to cancer cells via various mechanisms, researchers suggest that it could be an ideal agent for ovarian cancer treatment or an adjuvant agent in combination with other anticancer drugs [61].

The potential of rutin is well described in the literature, research indicates its suppression of numerous human cancers such as lung, prostate, colorectal, breast, liver, glioblastoma, melanoma, osteosarcoma, ovarian, leukemia, cervical, and pancreatic cancer via apoptosis induction, immunity enhancement, or cell migration knockdown, which leads to a significant reduction in the motility rate of cancerous cells [62]. Other flavonoids, such as apigenin and kaempferol, also are reported to inhibit the growth of human cervical carcinoma cells (HeLa and SiHa) through apoptotic pathways [63,64,65]. Studies suggest that these compounds may be useful adjuvant therapeutic agents in the treatment of cervical cancer. In addition to anticancer activities, kaempferol and its glycosylated derivatives are cardioprotective, neuroprotective, anti-inflammatory, antidiabetic, antioxidant, antimicrobial, and antitumor [66,67].

Eriodictyol, in previous studies, revealed the antioxidant capacity from the positive regulation of antioxidant defenses. Thus, this flavonoid can preserve the mitochondrial function of the human hepatocellular cancer cell (HepG2), significantly decreasing intracellular ROS and lipid peroxidation [68]. Eriodictyol was also tested in a lung cancer cell line, inducing mitochondrial-mediated apoptosis and causing G2/M cell cycle arrest. Therefore, the inhibition of the m-TOR/PI3K/Akt signaling pathway was observed after treatment with eriodictyol [69].

Apigenin has been shown to have strong therapeutic potential against a significant number of diseases [70]. The current literature data show that apigenin has many beneficial effects, including biological organ protection (heart, brain, liver, lung), hypotension, hypoglycemia, lipid-lowering effect, antioxidation, anti-inflammation, anti-osteoporosis, and immune regulation [71]. Many cancer studies describe the action of apigenin in cell cultures *in vitro,* as well as animal models *in vivo*. Among the therapeutic effects described in the suppression of various types of cancer carried out in multiple animal models of cancer are: liver, prostate, pancreatic, lung, nasopharyngeal, skin, colon, colorectal, colitis-associated carcinoma, head and neck squamous cell carcinoma, leukemia, renal cell carcinoma, Ehrlich ascites carcinoma, and breast cancer were designed to evaluate the chemopreventive property of this phytocompound [1].

Based on these data, it is possible to conclude that *F. platyphylla* extract, because it contains compounds such as rutin, quercetin derivatives, and glycosylated flavonoids, has the potential to exert anticancer effects through several molecular mechanisms. Among these, the restoration of p53 protein expression and the induction of apoptosis [56,57,58] stand out, as well as the stimulation of the immune response or the inhibition of cell migration, resulting in a significant reduction in cancer cell motility [62], in addition to its antioxidant action, promoting the neutralization of reactive oxygen species and, consequently, minimizing DNA damage and processes related to carcinogenesis [59]

Although the flavonoid aglycones eriodictyol, apigenin, taxifolin, and quercetin were not directly identified in the analyzed extract, their glycosylated derivatives were detected. These aglycones are intended to highlight the biological effects that may be related to the identified flavonoids, considering that such effects are often attributed to the aglycones released after metabolization or enzymatic hydrolysis. From a pharmacological perspective, flavonoid glycosides may offer advantages over their aglycones, since glycosylation can increase solubility in aqueous media, reduce toxic side effects, and improve specificity of action, favoring bioavailability and cellular targeting [72,73]. Cytotoxicity tests are essential to assess the safety and efficacy of plant products, as they determine their impact on cell viability in different cell types. It is crucial to include both cancerous and non-cancerous cells in the studies to identify the selectivity of the extracts, which helps to minimize unwanted side effects and ensure that treatments reach tumor cells without causing damage to normal cells [74].

In this context, researchers evaluated the cytotoxic effects of different extracts on non-cancerous cell lines, such as MDCK II (dog kidney epithelial cells) and RPE1 (human retinal epithelial cells). The focus of the evaluation was the impact on cell viability and morphology of these cells after the application of four different extracts (A, B, C, and D). The composition of extracts A, B, and C, rich in flavonoid glycosides and phenolic acids, contributed to their selectivity and safety in relation to non-cancerous cells, while extract D, with its higher concentration of aglycones, demonstrated more pronounced cytotoxicity [74].

In the *F. platyphylla* extract, 15 molecular structures were annotated, as shown in Table 1, demonstrating the presence of phenolic compounds such as chlorogenic acids and glycosylated flavonoids, belonging to the flavone and flavonol subclasses. Notable among these are rutin and quercetin derivatives, frequently mentioned in the literature for their anticancer effects, including the restoration of p53 protein expression and the induction of apoptosis in HPV-infected cells [56,57,58].

It is important to emphasize that this evidence is based on *in vitro* studies, which impose limitations on the extrapolation of the results to clinical settings. Therefore, we recognize the need for further investigation, including *in vivo* assays and more in-depth mechanistic studies, to validate and broaden the understanding of the observed effects.

## 3. Materials and Methods

### 3.1. Material Collection

*Fridericia platyphylla* (Cham.) L. G. Lohmann leaves were collected in Sant’Ana da Serra in João Pinheiro, Minas Gerais, Brazil (coordinates 17°4404500 S, 46°1004400 W), in February 2021. The plant was identified by the botanist Dr. Ana Maria Cristina Teixeira Braga at the José Badine Herbarium of the Federal University of Ouro Preto (Voucher 17.935). The access to the botanical material was registered in the National System for the Management of Genetic Heritage and Associated Traditional Knowledge (SisGen A4551DE4).

### 3.2. Preparation of Ethanolic Extract

The leaves were dried in an oven (Hexasystems, SSDic São Paulo, Brazil) at a temperature of 40 to 50 °C and crushed in knife mills (Trapp, Santa Catarina, Brazil). For the extraction of 150 g of dried leaves, a total volume of 4.10 mL of 70% ethanol (Êxodo Científica, Campinas, Brazil) was used, in a 7:3 ratio. The extraction was performed through exhaustive percolation, with 24 h extraction cycles, during which 216 mL of 70% ethanol was added in each cycle. Then, the extracting liquid was filtered and subjected to rotatory vacuum evaporation in a system (vacuum pump, model TE-058, Tecnal, Piracicaba, Brazil; Rotaevaporator, model 802, Fisatom, São Paulo, Brazil; ultrathermostatic bath, model SSDu-20L, SolidSteel, Piracicaba, Brazil) at a temperature below 40 °C and subsequently freeze-dried (Lyophilizer Model K105, Liotop, São Carlos, Brazil) for 48 h at a temperature of −95 °C and pressure of 12 µHg. At the end of the process, the ethanolic extract then presented a mass equal to 29.35 g. The yield was 19.56%.

### 3.3. Liquid Chromatography-Mass Spectrometry (LC-MS) Analysis

Chemical analysis for metabolite annotation by MS/MS data processing with molecular networking was performed by liquid chromatography-electrospray ionization ion trap mass spectrometry (LC-ESI-IT-MS) with a spectrometer (amaZon SL Bruker Daltonics^®^, Billerica, MA, USA). The chromatographic analysis was performed on a Luna 5 µm C18 100 Å column (250 × 4.6 mm, Phenomenex, Torrance, CA, USA), with HPLC Prominence Shimadzu^®^. The binary gradient mobile phase consisted of 0.1% formic acid (Sigma-Aldrich, St. Louis, MO, USA) in water (solvent A) and 0.1% formic acid in methanol (Sigma-Aldrich, St. Louis, LO, USA) (solvent B). Samples (1 mg mL^−1^) were eluted from the analytical column with a 40 min gradient ranging from 5 to 100% solvent B at a constant 1 mL min^−1^ flow rate. The injection volume was 2 µL. Column compartment temperature set to 40 °C.

Data acquisition was performed in both negative and positive modes, with fragmentation in multiple stages (MS^2^ and MS^3^), using a Data-Dependent Acquisition (DDA) method, according to the following parameters: nebulization gas pressure, 50.0 psi; capillary temperature, 300 °C; transfer capillary input voltage, 4500 V; desolvation gas, nitrogen (N_2_), flow 10 L.min^−1^; collision gas, helium (He); range acquisition, *m*/*z* 50–1200. Raw data were analyzed and converted for mzML format using Data Analysis 4.3 software (Bruker, MA, USA) [75].

### 3.4. MS/MS Data Processing and Classical Molecular Networking

The classical molecular network was created using the online workflow on the Global Natural Products Social Molecular Networking (GNPS) platform [76]. The data were filtered by removing all MS/MS fragment ions within ±17 Daltons (Da) of the precursor *m*/*z*. The MS/MS spectra were window filtered by choosing only the top 6 fragment ions in the ±50 Da window across the spectrum. The precursor ion and MS/MS fragment ion mass tolerances were adjusted to 0.5 Da. A network was created where edges were filtered to have a cosine value above 0.7 and more than four matching peaks. The maximum size of a molecular family was defined as 100.

The spectra were then searched against the GNPS spectral libraries. All matches maintained between network and library spectra needed a score above 0.7 and at least four matching peaks [76]. The network visualization was performed using the Cytoscape 3.9.1 software (National Institute of General Medical Sciences—NIGMS, Bethesda, MD, USA) [77]. In order to eliminate the contaminants, a blank injection was uploaded as a separate group in molecular network workflow, and the nodes (gray color) corresponding to the blank were excluded from the results. The analysis of molecular networks can be accessed on GNPS platform, and the dataset is available on MSV000096552 [78].

### 3.5. Anticancer Assays

#### 3.5.1. Cell Culture

HeLa and SiHa human cervical cancer cell lines were maintained in a monolayer culture in Dulbecco’s Modified Eagle’s Medium (DMEM) with 10% Fetal Bovine Serum (FBS) (*v*/*v*) and 1% penicillin/streptomycin (50 U mL^−1^/50 μg mL^−1^). The culture flasks were maintained at 37 °C in an atmosphere of 5% CO_2_ and 95% humidity. Cells displaying exponential growth were detached from the culture flasks with trypsin and seeded at the density required for the experiment.

#### 3.5.2. MTT Cytotoxicity Assays

Cell viability was assessed using the MTT [3-(4,5-dimethylthiazol-2-yl)-2,5-diphenyltetrazolium bromide] assay, where viable cells are detected by their ability to convert MTT into insoluble formazan crystals, as previously described [79]. Cervical cancer cells were seeded on a 96-well plate at a density of 1 × 10^4^ cells per well with DMEM containing 10% FBS and incubated under the previous conditions. The following day, after cell adhesion, the medium was replaced with different concentrations of samples (20–150 µg mL^−1^), or cisplatin (CPT) 5 µg mL^−1^, and cells were incubated for 24, 48, or 72 h. Blank wells (medium + compounds without cells) were included for the highest concentration of each sample. The vehicle (medium + dimethyl sulfoxide) was tested at the highest concentration (0.15%) used in the experiment and did not interfere with the activity of the samples. After the incubation period, the treatment was removed, the wells were washed with phosphate-buffered saline (PBS buffer, pH 7.2), and then a solution of medium containing 0.5 mg mL^−1^ of MTT reagent was added to each well. The plate was incubated under the previous conditions for 3 h. Then, the MTT solution was discarded, and the dark blue formazan crystals were dissolved in ethanol. The absorbance was measured at 570 nm using a microplate spectrophotometer reader (Epoch^®^, BioTekInstruments, Winooski, VT, USA/Gen5 Software). The results were expressed as percentages of living cells compared to control cells (cells without treatment). Data was analyzed using One-Way ANOVA followed by post hoc comparisons (Dunnett’s test) with GraphPad Prism7 software [80]. For each sample, results represent the mean ± standard deviation of three independent experiments, with each experiment performed in triplicate. *p*-values less than 0.05 were considered statistically significant. The calculation of the IC_50_ was performed by transforming the values to logarithms and applying the curve for nonlinear regression using the GraphPad Prism 7 [80] software (GraphPad Software^®^, San Diego, CA, USA) [81].

#### 3.5.3. Wound-Healing Assay

Cells (1 × 10^5^ cells/well) were plated in DMEM with FBS 10% in 96-well plates and incubated (atmosphere of 5% CO_2_ and 95% humidity, at 37 °C) for 24 h. After confirming the formation of a complete monolayer, the cells were wounded by scratching lines with a standard 10 μL plastic tip. Then, the culture medium was removed, the wells were washed with PBS buffer (to remove debris), and 180 µL of culture medium was added. Then, the plate was covered and sealed on the sides with parafilm, and each well was photographed using an inverted microscope (Opticam O500i) equipped with a camera (NA 0.30, WD 72) and a 10× objective. Afterward, the plate was returned to the laminar flow, and 20 µL of FAB (10× concentrated) was added to the wells, to obtain final concentrations of 20, 40, 60, and 100 µg mL^−1^, or 20 µL of cisplatin (CPT) (10× concentrated) to obtain concentration of 5 µg mL^−1^, or it was placed the culture medium (control), and the plate was incubated again. These photographic records correspond to time zero (T_0_). After 24 h, 48 h, and 72 h of treatment, the wells were photographed again (T_F_) to determine the migration and cell movement throughout the wound area. Each experiment was performed in triplicate.

The images were analyzed using the ImageJ software, version 1.54V (Wayne Rasband, National Institute of Health, Bethesda, MD, USA), which allowed the measurement of the area of each groove. The area of the grooves was analyzed at times: 0 h, 24 h, 48 h, and 72 h. The percentage of scratch closure was quantified by the percentage variation between the area in T_0_ and the area at T_F_ (corresponding to 24 h, 48 h, or 72 h), according to the formula: %Closure = [(T_0_ − T_F_/T_0_) × 100]. The means and standard deviations of the results were determined. The means of the treated groups were compared with the means of their respective control, determining the closure effect through the decrease in the percentage, as described in the formula above.

## 4. Conclusions

The results obtained show that the crude extract of *F. platyphylla* leaves (FAB) can act as an antiproliferative and anti-migratory agent, since in the HeLa and SiHa cell lines it reduced cell viability in a concentration- and time-dependent manner, as well as being effective in inhibiting migratory capacity. In addition, they highlight the contributions of phenolic acid derivatives, flavonoid glycosides, and flavone aglycones present in this extract, acting synergistically in promoting antitumor activity. Although the presence of brachydins is commonly associated with this activity in *F. platyphylla* flowers and roots, this study demonstrates that, even in the absence of these substances in the leaves, the other bioactive compounds are capable of exerting significant biological effects, reinforcing the therapeutic potential of the leaf extract. In this way, the findings with FAB open up promising prospects in the search for new bioactives and innovative therapeutic approaches for the treatment of cervical cancer. *In vivo* trials and further investigations into the mechanisms of action are essential to deepen and confirm the effects observed, since they will not only validate the efficacy and safety of the extract, but will also contribute to the development of new therapeutic strategies based on natural products.

## Figures and Tables

**Table 1:** Chemical constituents annotated in the hydroethanolic extract of *Fridericia platyphylla* leaves.

Peak	Retention Time (min)	Compound *	Stage	Fragments *m*/*z* (−)	Fragments *m*/*z* (+)	References
**1**	11.6	caffeoylquinic acid	MS^2^	353 ** [M − H]^−^; **191**; 179	-	[20,21,22]
**2**	14.2	feruloylquinic acid	MS^2^	369 ** [M − H]^−^; **193**; 174; 134	-	[23]
**3**	15.7	apigenin 6,8-*C*-dihexoside	MS^2^	593 [M − H]^−^; **473**; 385; 355	595 [M + H]^+^; **577**; 559; 529; 475; 457	[24]
**4**	16.8	apigenin 6-*C*-pentosyl-8-*C*-hexoside	MS^2^	563 [M − H]^−^; 545; 473; 443; **385**; 355	565 [M + H]^+^; 547; 529; **429**; 411	[24,25]
**5**	16.9	apigenin 6-*C*-hexosyl-8-*C*- pentoside	MS^2^	563 [M − H]^−^; 545; 473; **443**; 385; 355	565 [M + H]^+^; **547**; 529; 429; 411	
**6**	17.5	apigenin 6-*C*-hexosyl-8-*C*-deoxihexoside	MS^2^	577 [M − H]^−^; 487; **457**; 369; 339	579 [M + H]^+^; 561; 543; **443**	[26]
**7**	18.9	rutin	MS^2^	609 [M − H]^−^; 301 **	611 [M+ H]^+^_;_ 303 **	[27,28,29]
**8**	19.1	quercetin-*O*-hexoside	MS^2^	463 [M − H]^−^; 301 **	-	[30]
**9**	20.1	Kaempferol-*O*- deoxyhexosyl-*O*-hexoside	MS^2^	593 [M − H]^−^; 285 **	595 [M + H]^+^; 549; 449; **287**	[31]
**10**	20.5	quercetin-*O*-deoxyhexosyl-*O*-dihexoside	MS^2^	771 [M − H]^−^; 609; 301 **	-	[32]
**11**	21.3	quercetin-*O*- dimethoxycaffeoyl-*O*- hexosyl-*O*-hexoside	MS^2^	817 ** [M − H]^−^; 609; 591; 301 **	-	[32]
**12**	21.5	quercetin-*O*-feruloyl-*O*-deoxyhexosyl-*O*- hexoside	MS^2^	785 [M − H]^−^; 609; 591; 301 **	-	[32]
**13**	22.6	kaempferol-*O*-feruloyl-*O*-deoxyhexosyl-*O*- hexoside	MS^2^	769 [M − H]^−^; 593; 575; 285 **	-	[33]
**14**	23.8	3,4′-dimethoxy-quercetin	MS^2^	331 ** [M − H]^−^; 314; 299	-	[34]
**15**	25.5	tamarixetin	MS^2^	315 ** [M − H]^−^; 300	-	[34]

Caption: * identification attempt; In bold, the ion corresponding to 100% abundance. ** Corrected mass value: adjusted by ±2 Da due to instrument miscalibration.

**Table 2:** The extract IC_50_ value after 24–72 h treatment.

Cell Lines	24 h	48 h	72 h
HeLa	78.78 µg mL^−1^	59.52 µg mL^−1^	44.78 µg mL^−1^
SiHa	81.90 µg mL^−1^	50.01 µg mL^−1^	66.97 µg mL^−1^

IC_50_: half-maximal inhibitory concentration.

**Table 3:** Cytotoxic effect of *F. platyphylla* on HeLa and SiHa cell lines. The cells were treated with the extract in a 20–100 µg mL^−1^ concentration.

HeLa	% Closure [(T_0_ − T_F_/T_0_) × 100]		
	Mean ± SD		
Extract (µg mL^−1^)	24 h	48 h	72 h
CTRL^−^	47.94 ± 1.57	75.02 ± 4.78	83.22 ± 5.65
CPT 5	27.77 ± 9.57	35.80 ± 6.24	41.94 ± 2.88
FAB 20	49.48 ± 6.54	75.93 ± 4.90	89.93 ± 8.12
FAB 40	42.24 ± 6.90	62.77 ± 5.46	76.33 ± 5.23
FAB 60	44.50 ± 3.81	50.74 ± 4.82	56.43 ± 10.78
FAB 100	21.98 ± 7.35	22.28 ± 8.39	24.53 ± 9.42
**SiHa**		**% Closure [(T_0_ − T_F_/T_0_) × 100**	
		**Mean ± SD**	
**Extract (µg mL^−1^)**	**24 h**	**48 h**	**72 h**
CTRL^−^	38.00 ± 2.52	68.00 ± 4.61	89.00 ± 10.42
CPT 5	34.20 ± 1.10	60.40 ± 2.99	87.90 ± 6.16
FAB 20	42.31 ± 3.23	73.04 ± 5.61	96.38 ± 3.19
FAB 40	38.33 ± 3.21	45.10 ± 3.38	47.21 ± 3.74
FAB 60	29.16 ± 4.15	30.75 ± 3.44	32.60 ± 3.55
FAB 100	18.10 ± 2.84	19.19 ± 3.18	19.73 ± 2.72

SD: standard deviation; CTRL^−^: control negative; CPT 5 (1.5 µg mL^−1^): cisplatin.

## Data Availability

The original contributions presented in this study are included in the article/Supplementary Material. Further inquiries can be directed to the corresponding authors.

## Supplementary Materials

The following supporting information can be downloaded at: https://www.mdpi.com/article/10.3390/plants14172693/s1, Table S1. Results of the analysis via GNPS platform of the MS/MS data of the hydroethanolic extract from leaves of *Fridericia platyphylla*; Figures S1–S20. Mass spectra of the annotated compounds; Scheme S1–S20. Proposed fragmentation of the annotated compounds.
