# Supplementary material for: Antiproliferative and Anti-Migratory Activities of an Extract from Fridericia platyphylla Leaves and Its Molecular Profile
Source: Plants (Basel). 2025 Aug 28;14(17):2693. doi: 10.3390/plants14172693 (PMC12430664; doi:10.3390/plants14172693)
Supplement: Supplementary file 1 [file plants-14-02693-s001.zip › plants-3785939-supplementary.pdf]

plants MDPI

Supplementary Materials:

Table S1. Results of the analysis via GNPS platform of the MS/MS data of the hydroethanolic extract from leaves of *Fridericia platyphylla.*

#	Rt (min)	Compound	[M – H]⁻	Fragments MS/MS
**1**	11.5	caffeoylquinic acid	353	191, 179
**2**	15.3	tricin-*C*-hexoside	491	401; 371
**3**	14.3	feruloyl quinic acid	367	193, 174, 134
**4**	16.7	apigenin-*C*-hexoside	475	385; 355
**5**	15.7	apigenin-*C*-dihexoside	593	473; 385; 355
**6**	17.0	apigenin-*C*-pentosyl-C-hexoside	563	545; 473; 443; 385; 355
**7**	19.1	quercetin-*O*-hexoside	463	303
**8**	17.5	apigenin-*C*-hexosyl-*C*-deoxyhexoside	577	487; 457; 369; 339
**9**	20.3	kaempferol-*O*-hexoside	447	285
**10**	18.8	rutin	609	301
**11**	23.30	dihydrokaempferol-*O*-deoxyhexose-*O*-hexoside	593	287
**12**	20.6	quercetin-*O*-hexoside-*O*-hexoside-*O*-deoxyhexoside	771	609; 303
**13**	21.3	quercetin-*O*-dimethoxycaffeoyl-*O*-hexosyl-*O*-hexoside	815	609; 591; 303
**14**	21.6	quercetin-*O*-feruloyl-*O*-deoxyhexosyl-*O*-hexoside	785	609; 591; 303
**15**	26.35	quercetin-*O*- hexoside-*O*-deoxyhexoside-*O*- deoxyhexoside	755	609; 303

Rt: retention time.

Other compounds were also annotated via molecular networking analysis, as shown in Table S1. This was possible because of the grouping of the nodes that were before annotated via inspection of raw data, allowing the expansion of the annotation compounds. This was possible due to the similar fragmentation profile of the special metabolites.

A molecular networking to glycosylated flavonoids was observed. The molecular ion *m/z* 491, was associated with compound **2**, denominated Tricin-*C*-hexoside. Fragments ions *m/z* 401 and *m/z* 371 were observed, characteristic of *C*-glycosylated flavonoids. Tricin is a hydroxy-flavone and is common in plants.

Compound **4** is also a glycosylated flavonoid and was annotated as apigenin-*C*-hexoside. The losses of 90 and 120 were observed because of the fragment at *m/z* 385 and *m/z* 355.

A molecular networking to quercetin (*m/z* 303) derivatives was observed, this is a example that we can expand the annotation based on the analysis of the raw data (MS^2 and MS^3). From molecular ion *m/z* 755, and MS/MS fragmentation patterns *m/z* 609 and *m/z* 303, characteristics of the sugar portions, compound **14** was annotated as quercetin-*O*-hexoside-*O*-deoxyhexoside-*O*-deoxyhexoside.

It is also clear that there were nodes in the molecular network that did not correspond to the GNPS platform, and this does not mean that the substance is new, as the spectral libraries are not complete and have limitations but can work as a complementary tool in the chemical profile of complex matrix.

MS² spectra of compounds annotated in *Fridericia platyphylla* leaf extract in negative ionization mode

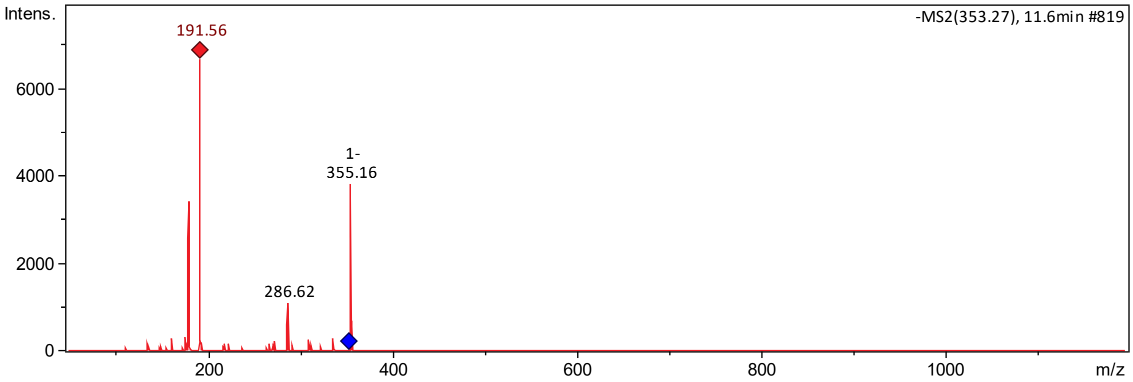

Figure S1. Mass spectrum of caffeoylquinic acid. *m/z:* 355: The compound was analyzed using a miscalibrated mass spectrometer, which resulted in a mass shift of approximately 2 Da.

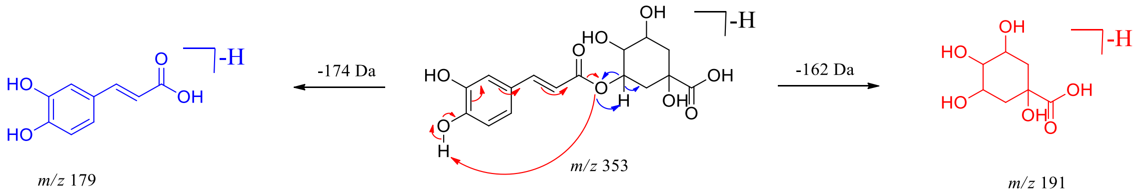

Scheme S1. Proposed fragmentation of caffeoylquinic acid in negative mode.

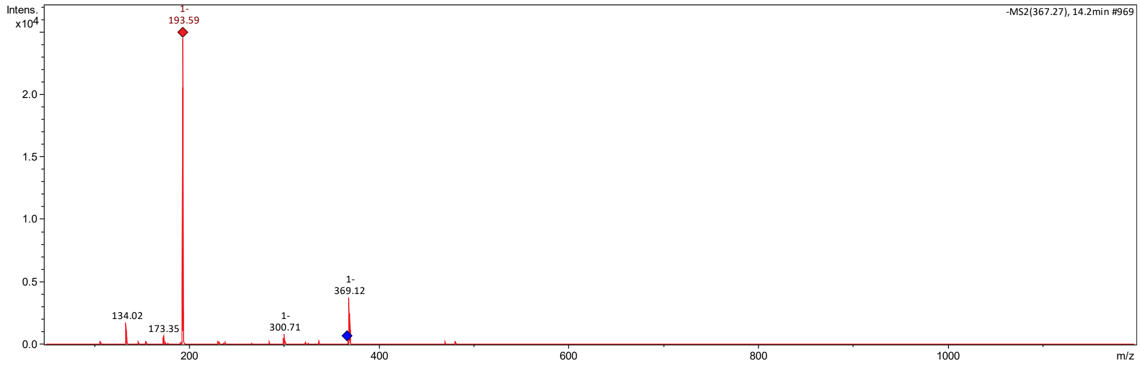

Figure S2. Mass spectrum of feruloylquinic acid. *m/z:* 367: The compound was analyzed using a miscalibrated mass spectrometer, which resulted in a mass shift of approximately 2 Da.

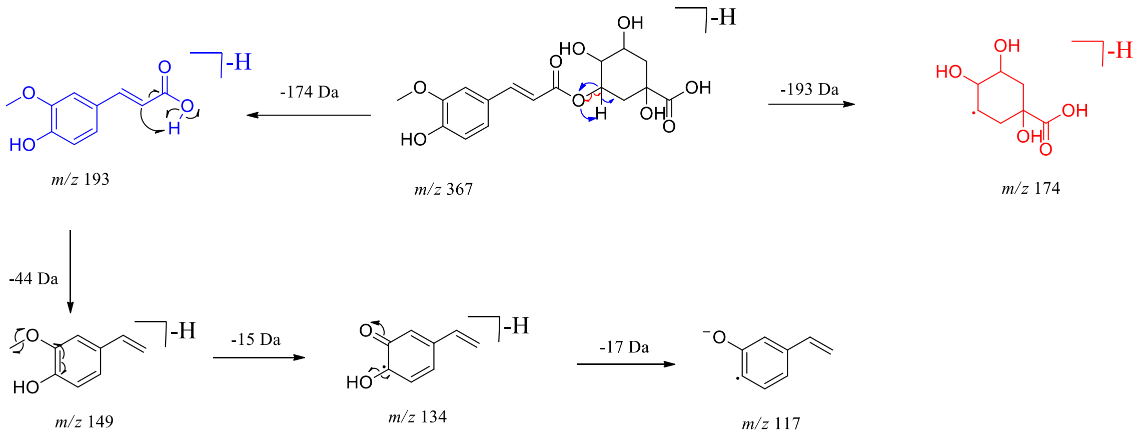

Scheme S2. Proposed fragmentation of feruloylquinic acid in negative mode.

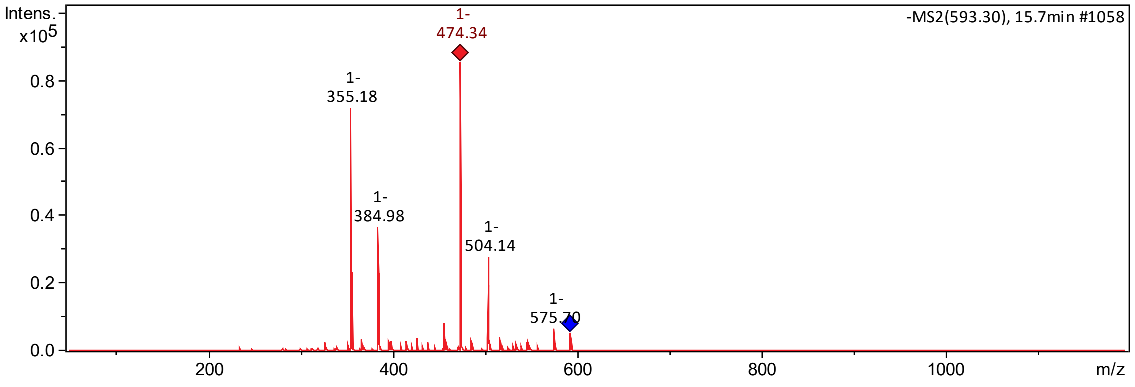

Figure S3. Mass spectrum of apigenin 6,8-C-dihexoside.

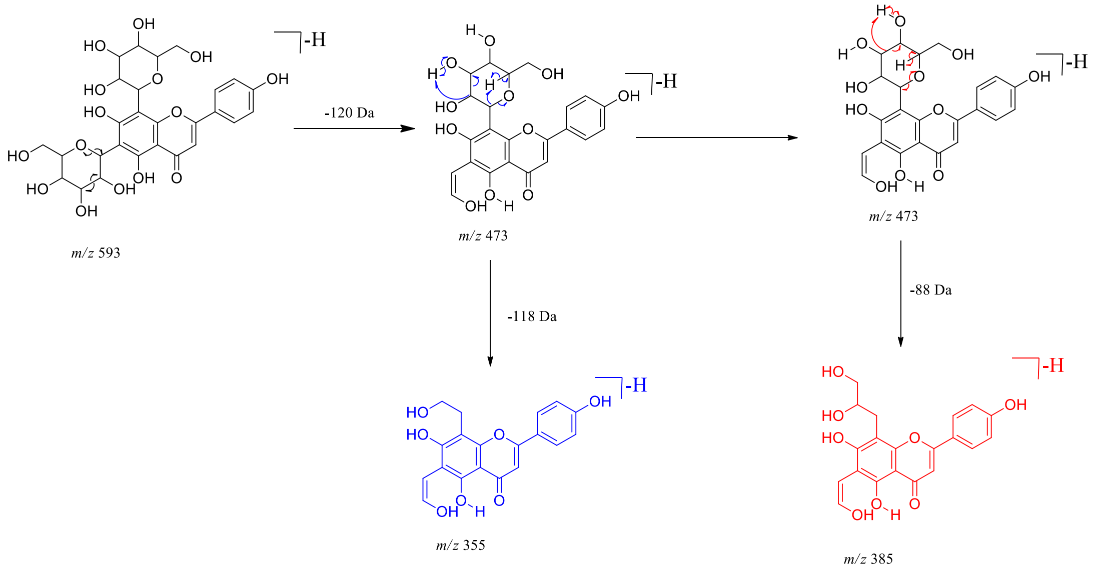

Scheme S3. Proposed fragmentation of apigenin 6,8-C-dihexoside in negative mode.

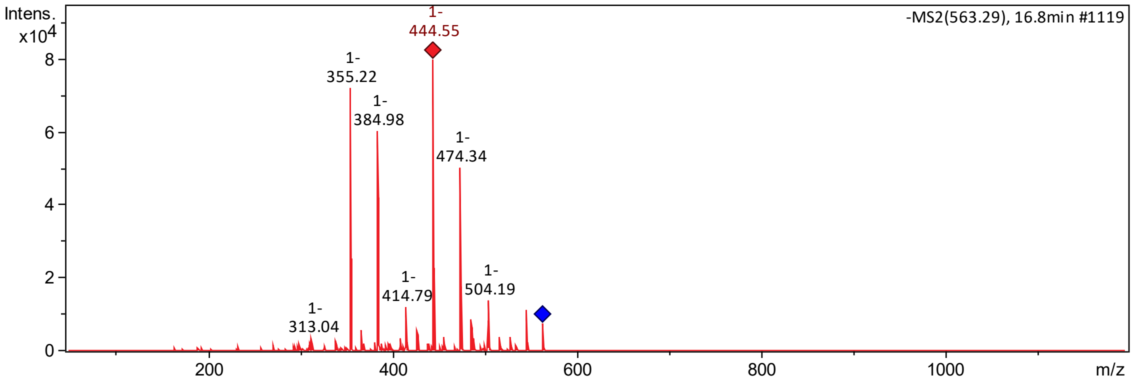

Figure S4. Mass spectrum of apigenin 6-*C*-pentosyl-8-*C*-hexoside.

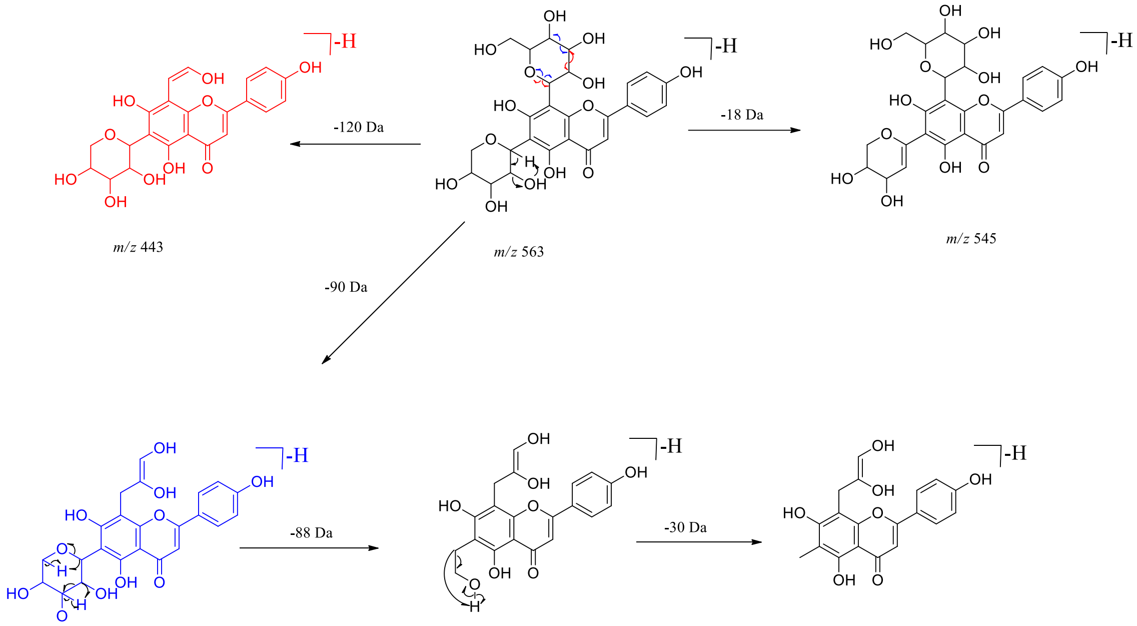

Scheme S4. Proposed fragmentation of apigenin 6-*C*-pentosyl-8-*C*-hexoside in negative mode.

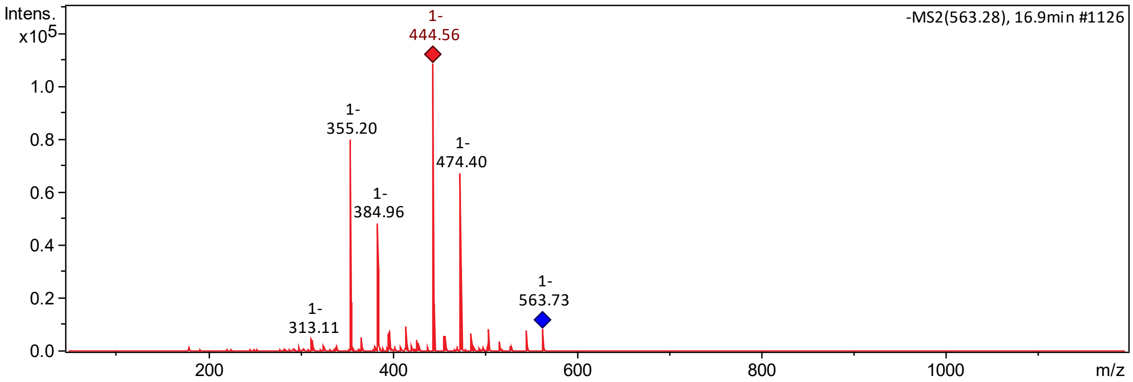

Figure S5. Mass spectrum of apigenin 6-*C*-hexosyl-8-*C*-pentoside.

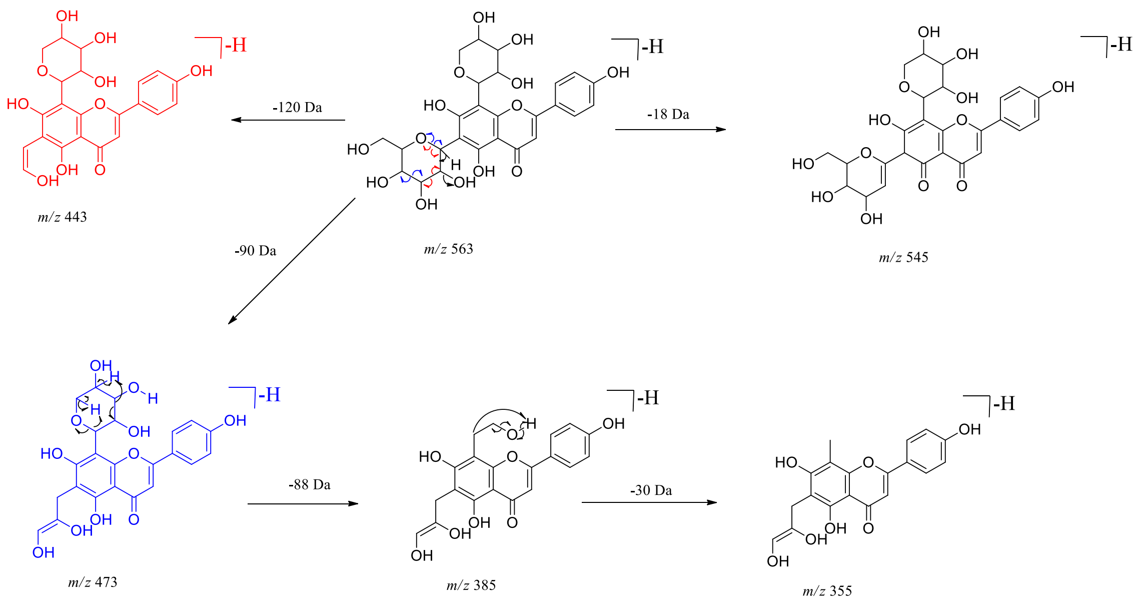

Scheme S5. Proposed fragmentation of apigenin 6-*C*-hexosyl-8-*C*-pentoside in negative mode.

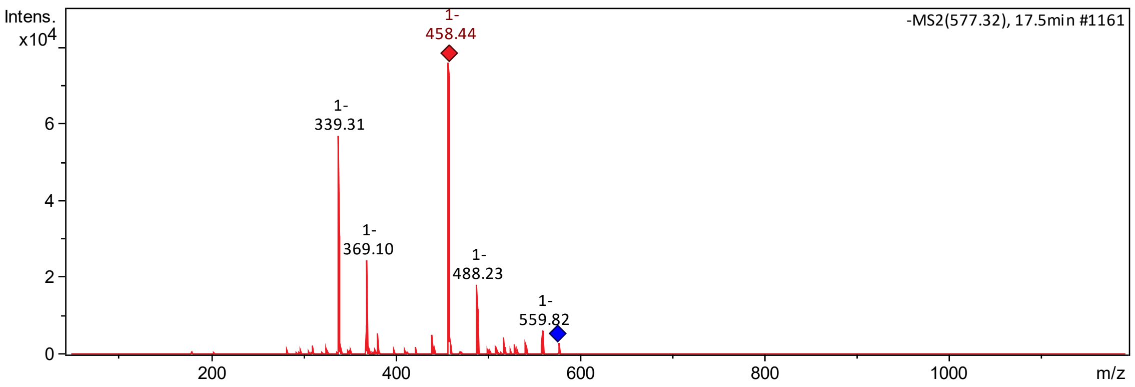

Figure S6. Mass spectrum of apigenin 6-*C*-hexosyl-8-*C*-deoxihexoside.

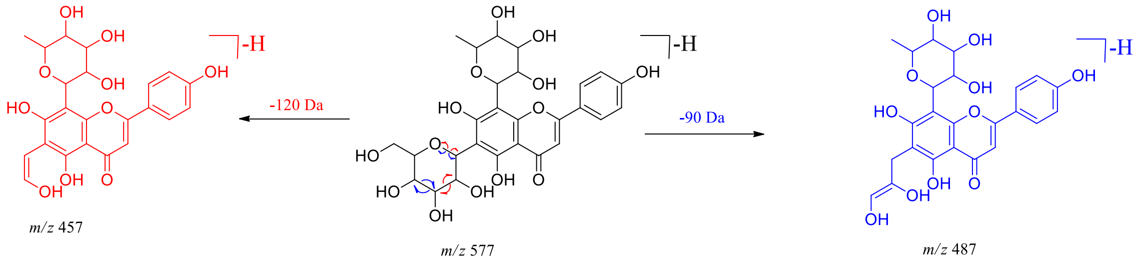

Scheme S6. Proposed fragmentation of apigenin 6-*C*-hexosyl-8-*C*-deoxihexoside in negative mode.

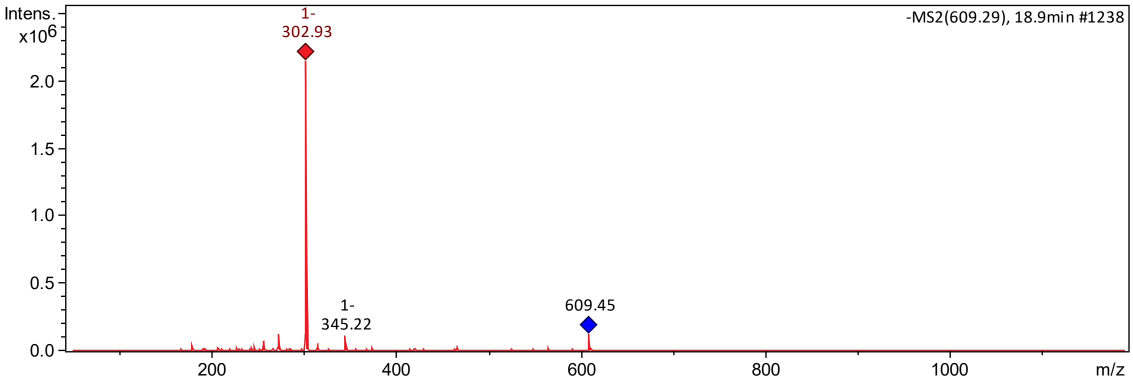

Figure S7. Mass spectrum of rutin. *m/z:* 303: The compound was analyzed using a miscalibrated mass spectrometer, which resulted in a mass shift of approximately 2 Da.

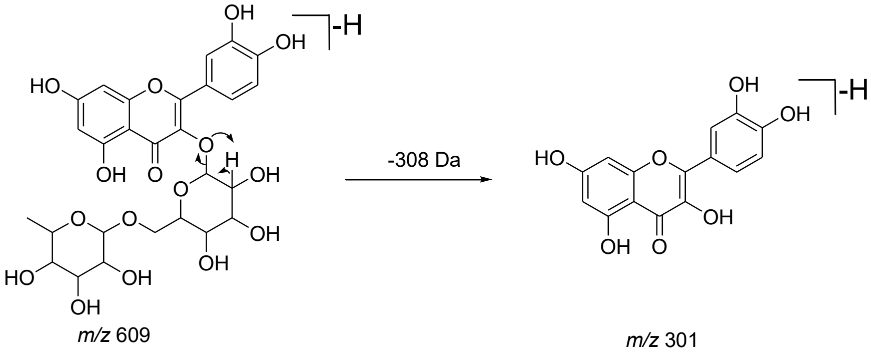

Scheme S7. Proposed fragmentation of rutin in negative mode.

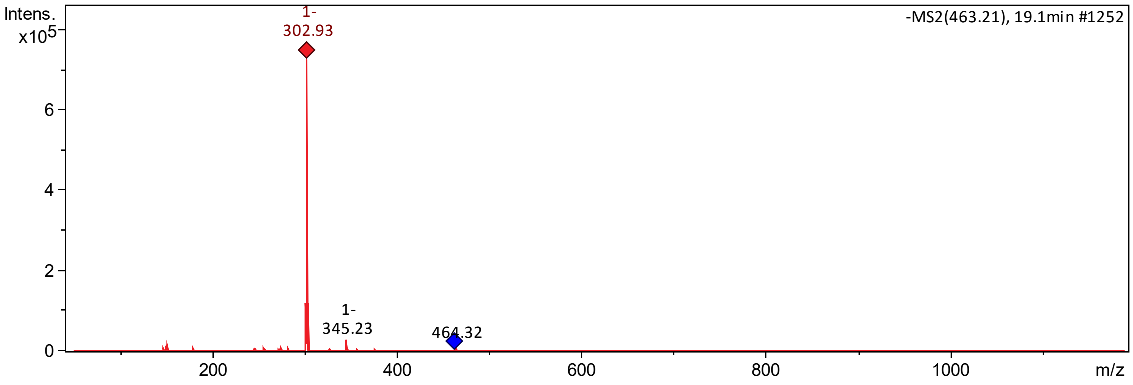

Figure S8. Mass spectrum of quercetin-*O*-hexoside. *m/z:* 303: The compound was analyzed using a miscalibrated mass spectrometer, which resulted in a mass shift of approximately 2 Da.

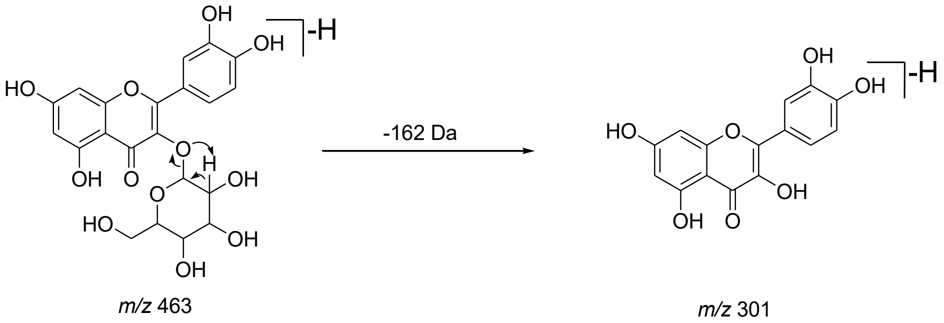

Scheme S8. Proposed fragmentation of quercetin-*O*-hexoside in negative mode.

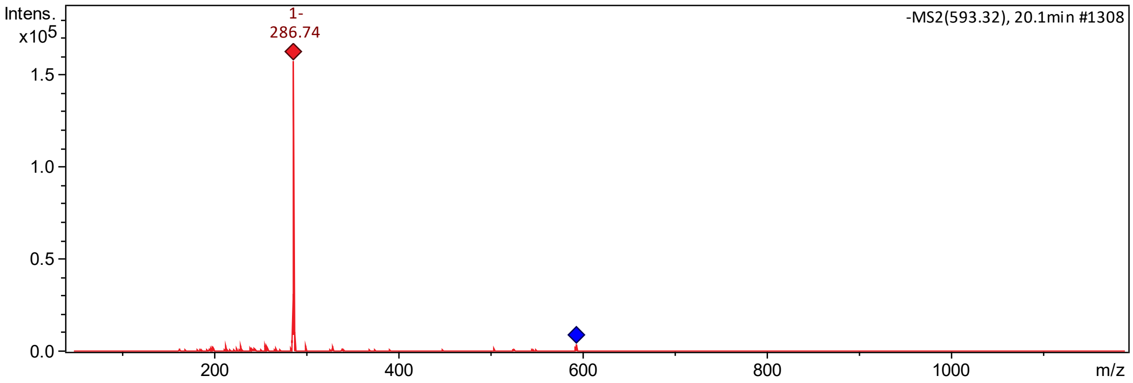

Figure S9. Mass spectrum of kaempferol *O*-deoxyhexosyl-*O*-hexoside. *m*/*z*: 287: The compound was analyzed using a miscalibrated mass spectrometer, which resulted in a mass shift of approximately 2 Da.

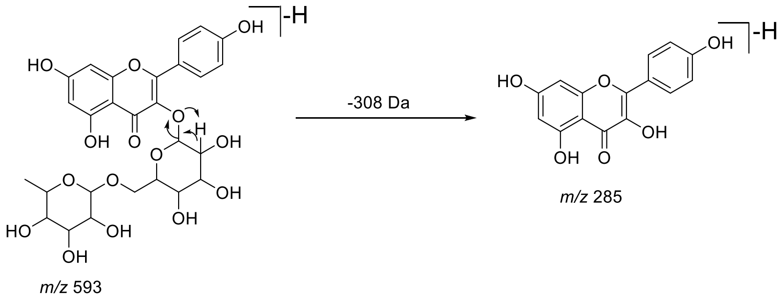

Scheme S9. Proposed fragmentation of kaempferol *O*-deoxyhexosyl-*O*-hexosidein negative mode.

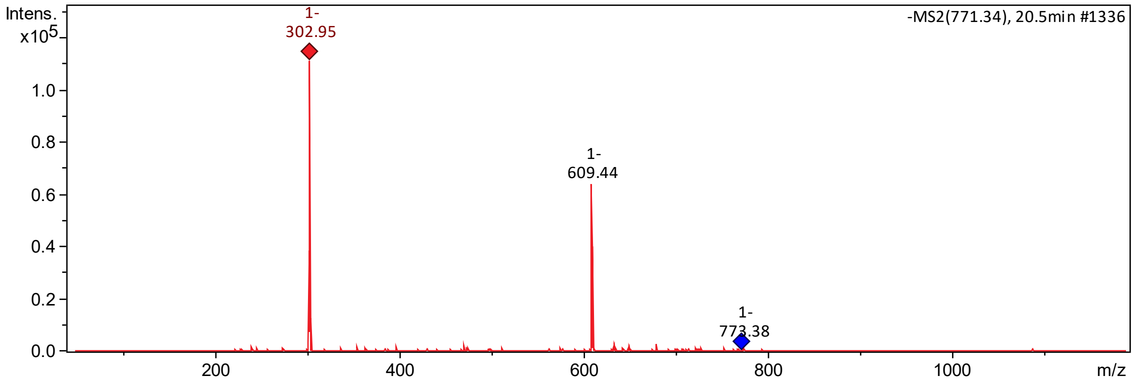

Figure S10. Mass spectrum of quercetin- *O*-deoxyhexosyl-*O*-dihexoside.*m*/*z*: 303: The compound was analyzed using a miscalibrated mass spectrometer, which resulted in a mass shift of approximately 2 Da.

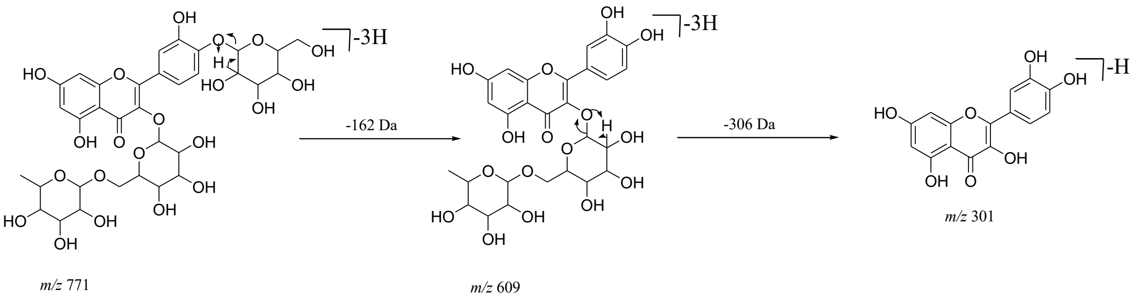

Scheme S10. Proposed fragmentation of quercetin- *O*-deoxyhexosyl-*O*-dihexoside in negative mode.

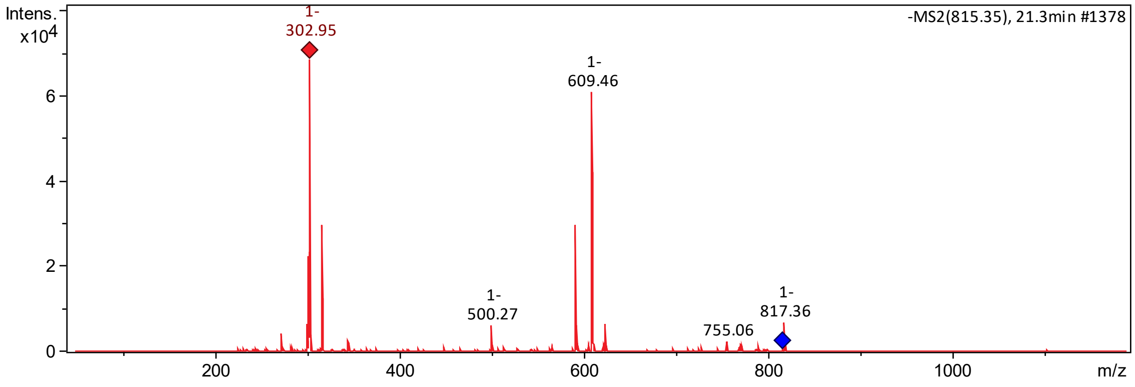

Figure S11. Mass spectrum of quercetin - *O*-dimethoxycaffeoyl-*O*-hexosyl-*O*-hexoside. *m/z:* 817 and *m/z* 303: The compound was analyzed using a miscalibrated mass spectrometer, which resulted in a mass shift of approximately 2 Da.

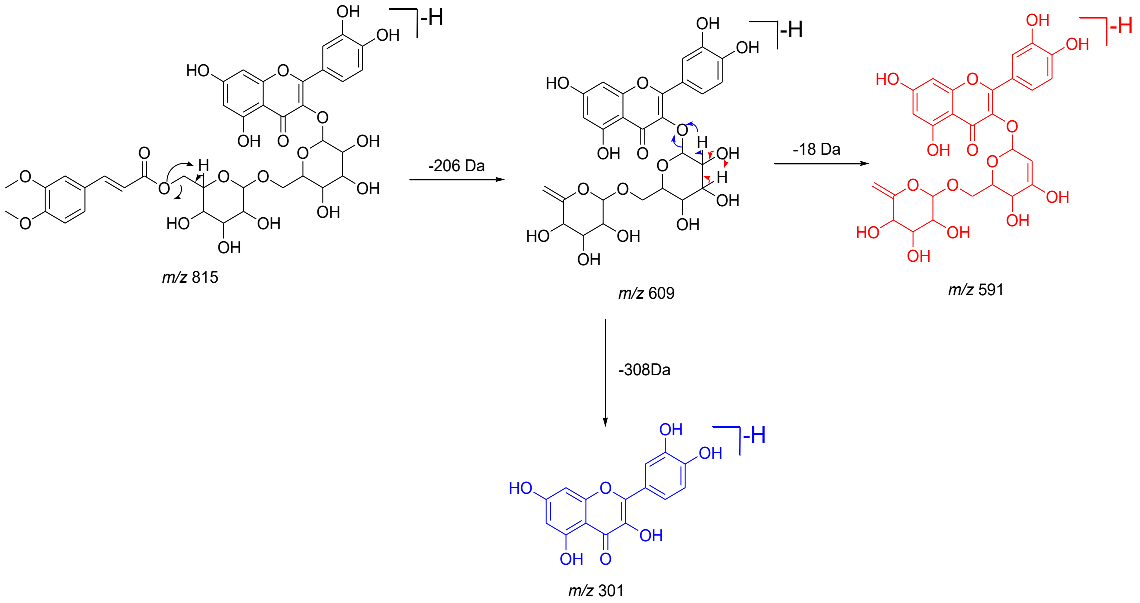

Scheme S11. Proposed fragmentation of quercetin -*O*-dimethoxycaffeoyl-*O*-hexosyl *O*-hexoside in negative mode.

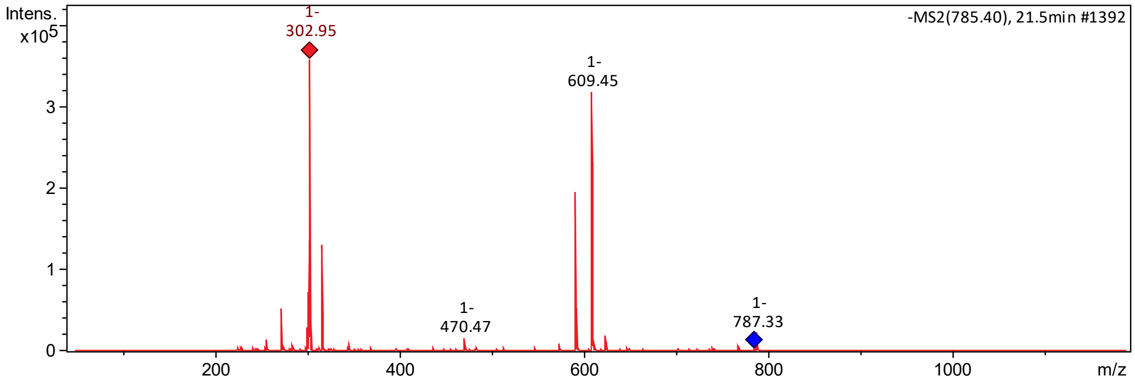

Figure S12. Mass spectrum of quercetin-*O*-feruloyl-*O*-deoxyhexosyl-*O*-hexoside. *m/z* 303: The compound was analyzed using a miscalibrated mass spectrometer, which resulted in a mass shift of approximately 2 Da.

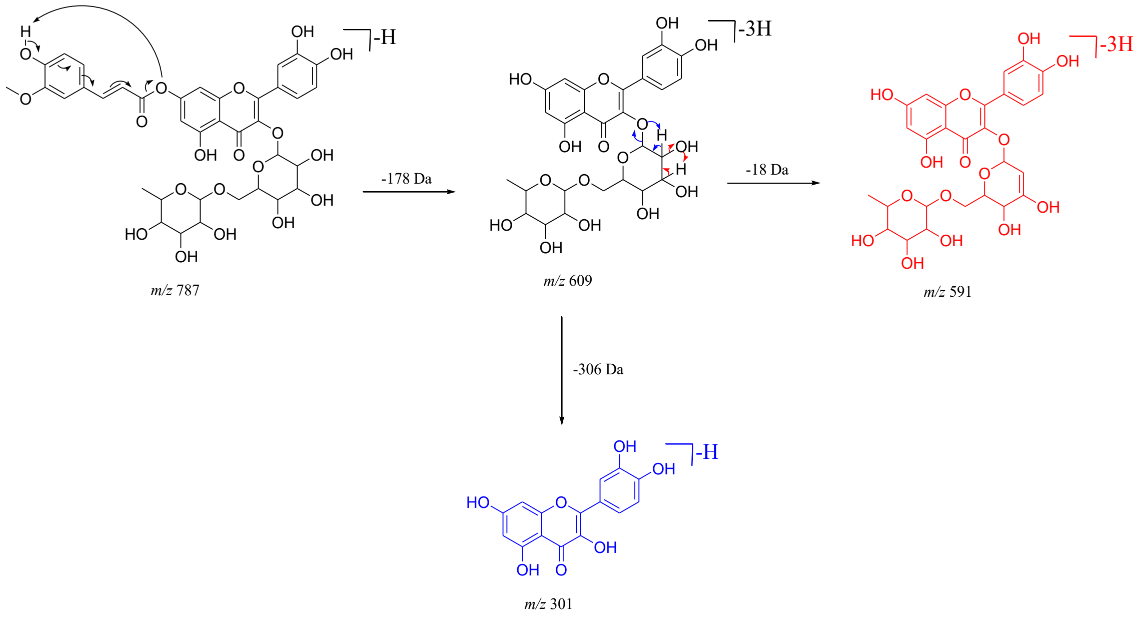

Scheme S12. Proposed fragmentation of quercetin-*O*-feruloyl-*O*-deoxyhexosyl-*O*-hexoside in negative mode.

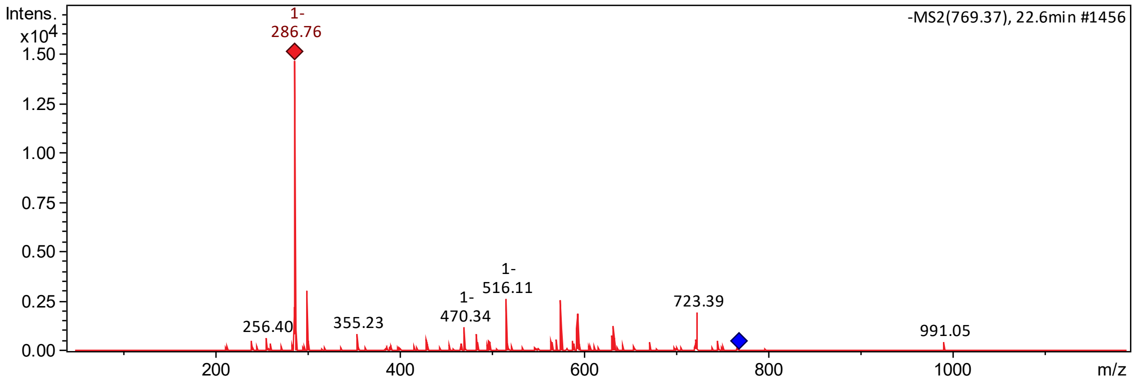

Figure S13. Mass spectrum of kaempferol-*O*-feruloyl-*O*-deoxyhexosyl-*O*-hexoside. *m/z:* 287: The compound was analyzed using a miscalibrated mass spectrometer, which resulted in a mass shift of approximately 2 Da.

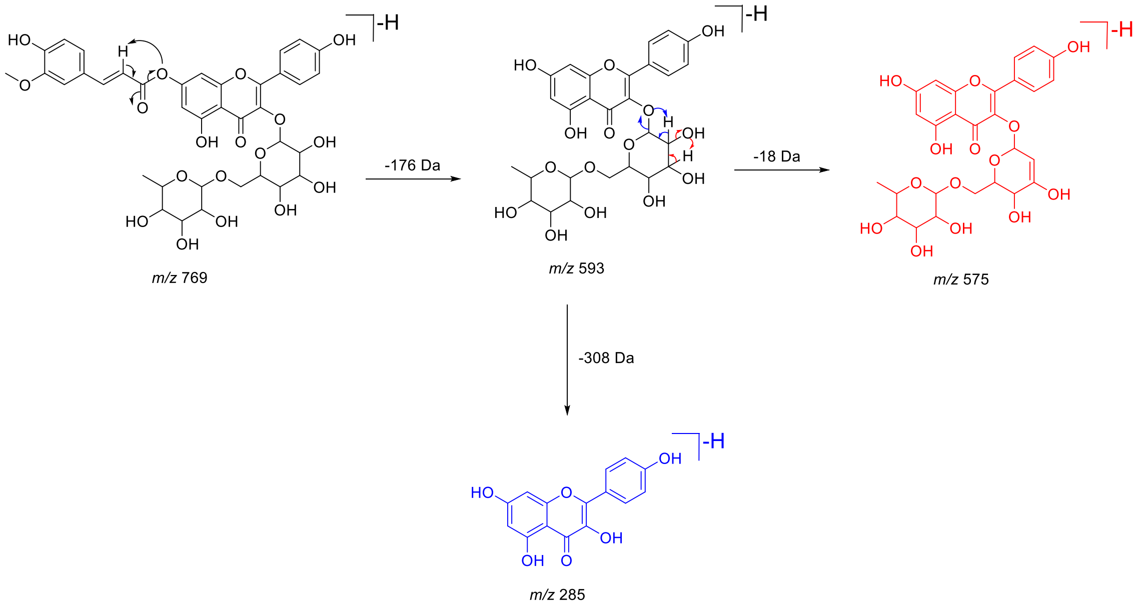

Scheme S13. Proposed fragmentation of kaempferol-*O*-feruloyl-*O*-deoxyhexosyl-*O*-hexoside.

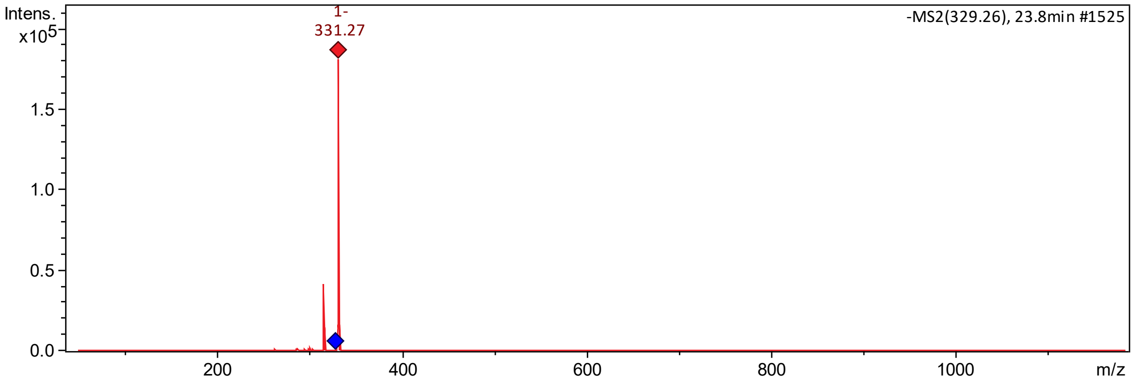

Figure S14. Mass spectrum of 3,4′- dimethoxy-quercetin. *m/z:* 331: The compound was analyzed using a miscalibrated mass spectrometer, which resulted in a mass shift of approximately 2 Da.

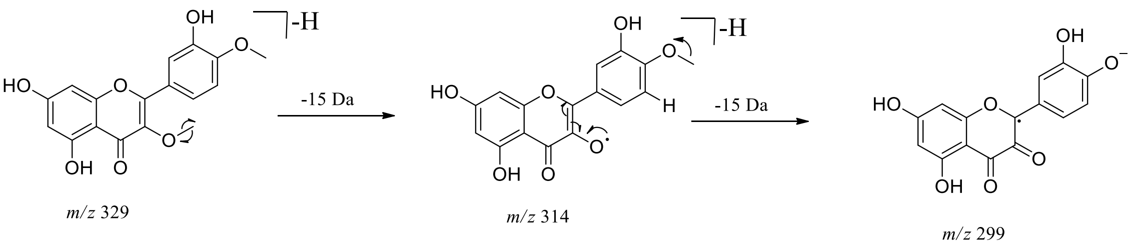

Scheme S14. Proposed fragmentation of 3,4′- dimethoxy-quercetin in negative mode.

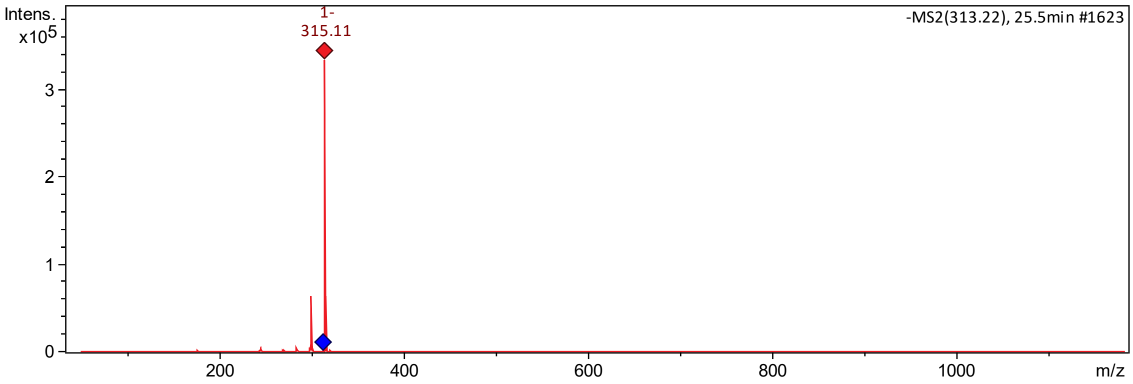

Figure S15. Mass spectrum of tamarixetin. *m/z:* 313: The compound was analyzed using a miscalibrated mass spectrometer, which resulted in a mass shift of approximately 2 Da.

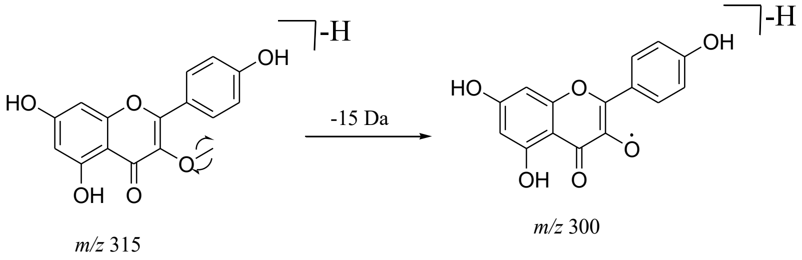

Scheme S15. Proposed fragmentation of tamarixetin in negative mode.

MS² spectra of compounds annotated in *F. platyphylla* leaf extract in positive ionization mode

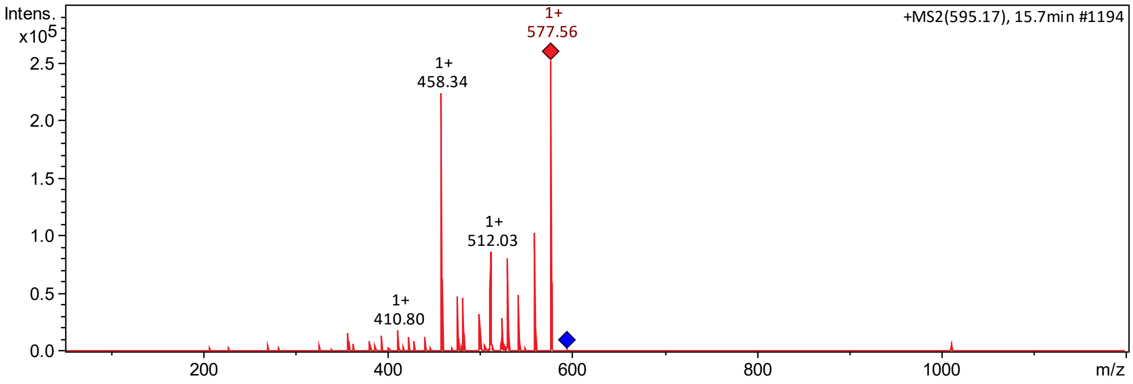

Figure S16. Mass spectrum of apigenin 6,8-*C*-dihexoside.

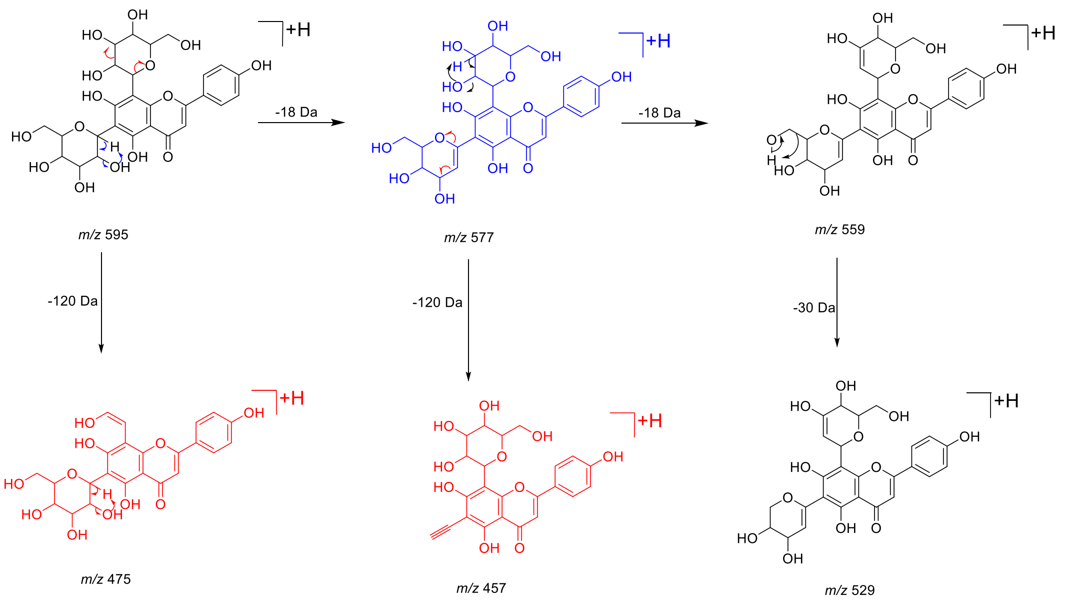

Scheme S16. Proposed fragmentation of apigenin 6,8-*C*-dihexoside in positive mode.

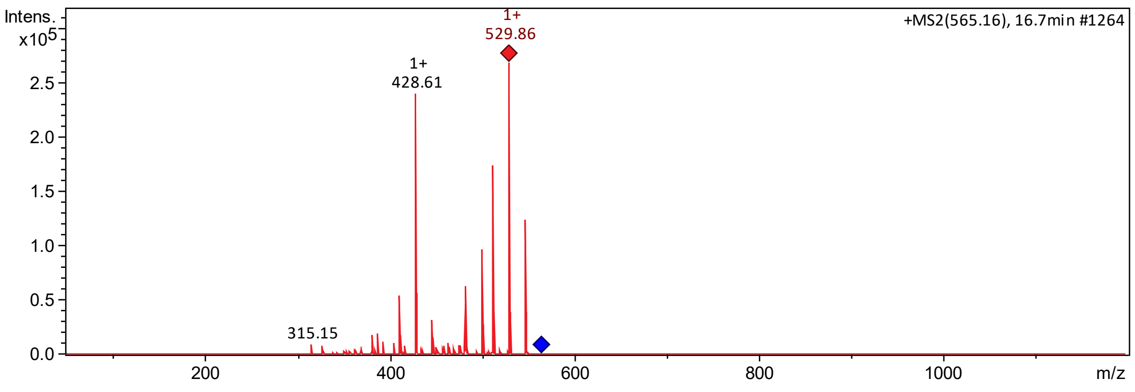

Figure S17. Mass spectrum of apigenin 6-*C*-pentosyl-8-*C*-hexoside.

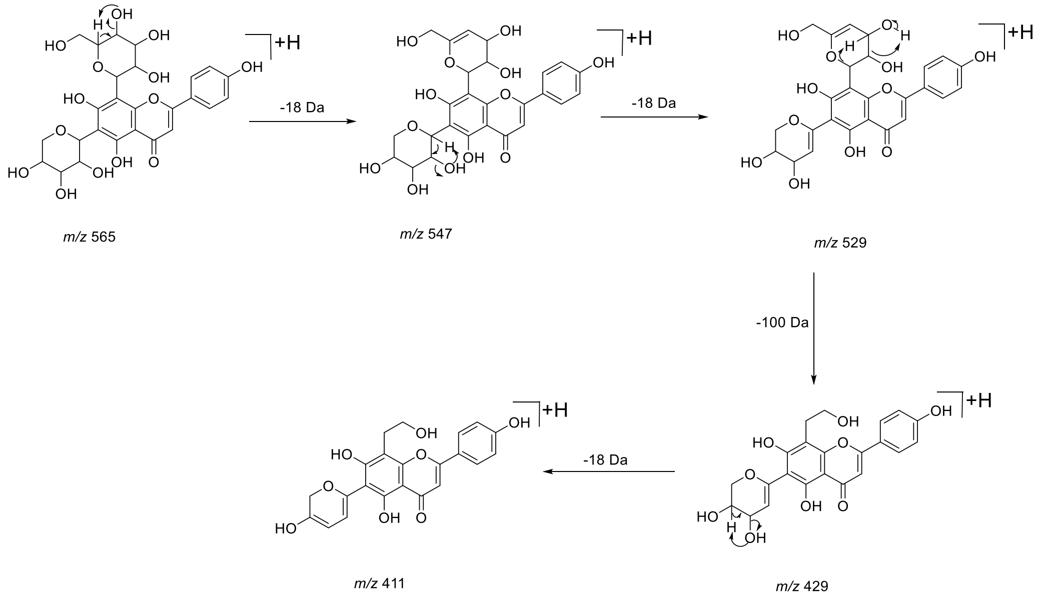

Scheme S17. Proposed fragmentation of apigenin 6-*C*-pentosyl-8-*C*-hexoside in positive mode.

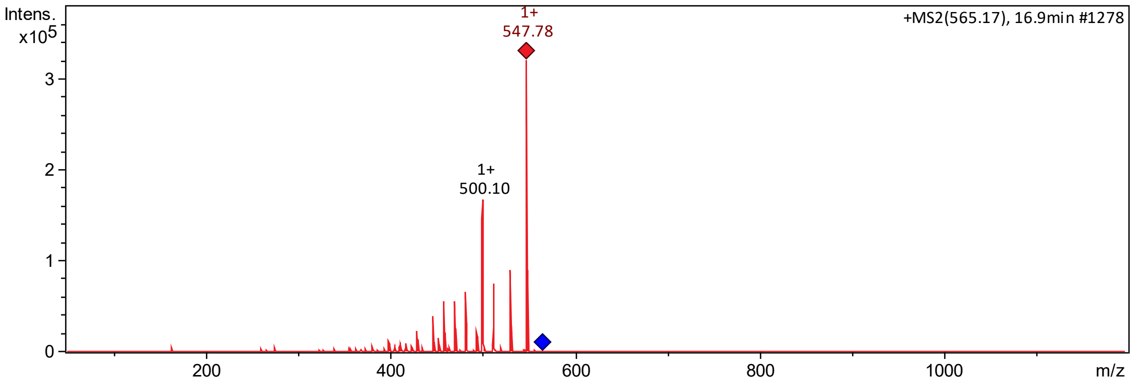

Figure S18. Mass spectrum of apigenin 6-*C*-hexosyl-8-*C*-pentoside.

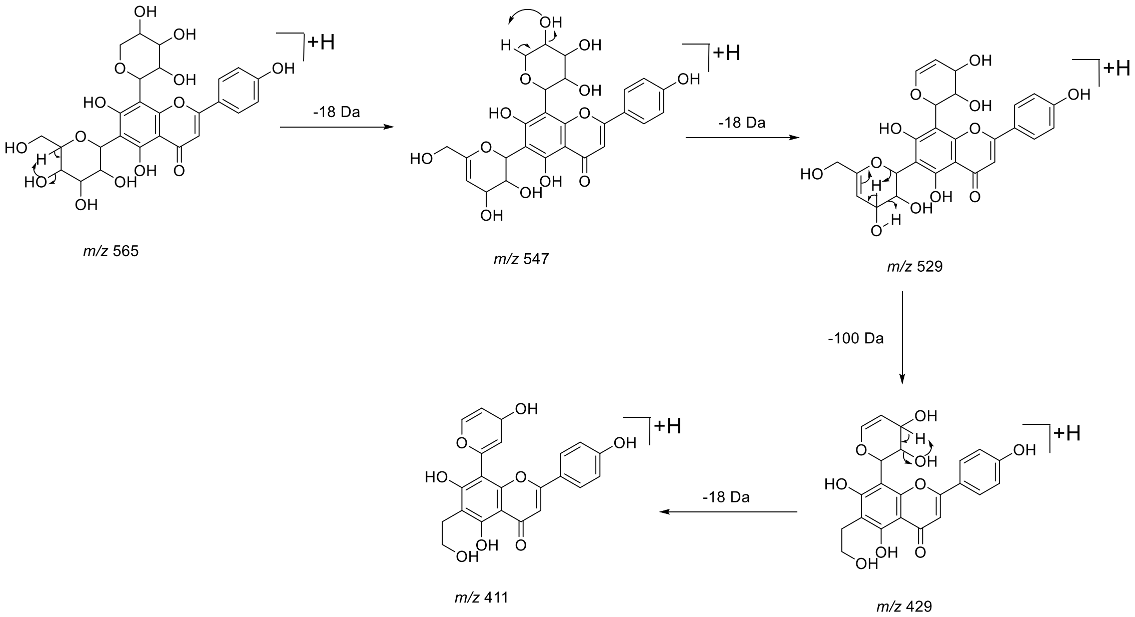

Scheme S18. Proposed fragmentation of apigenin 6-*C*-hexosyl-8-*C*-pentoside in positive mode.

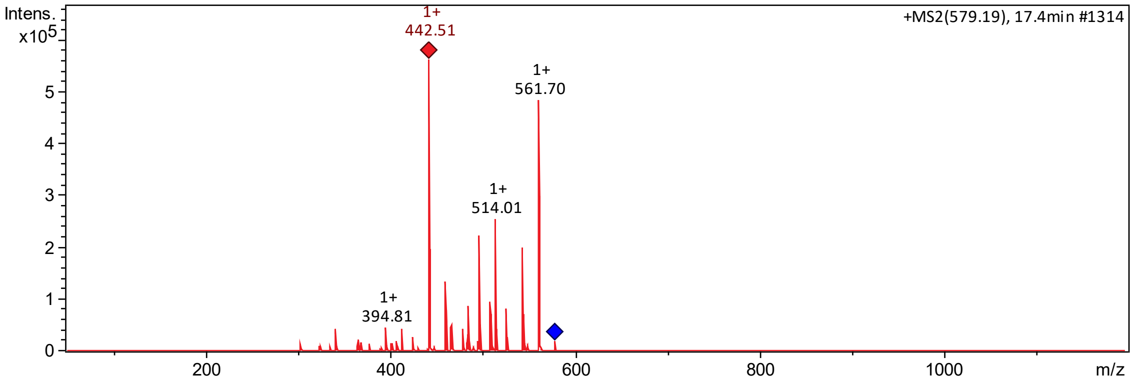

Figure S19. Mass spectrum of apigenin 6-*C*-hexosyl-8-*C*-deoxihexoside.

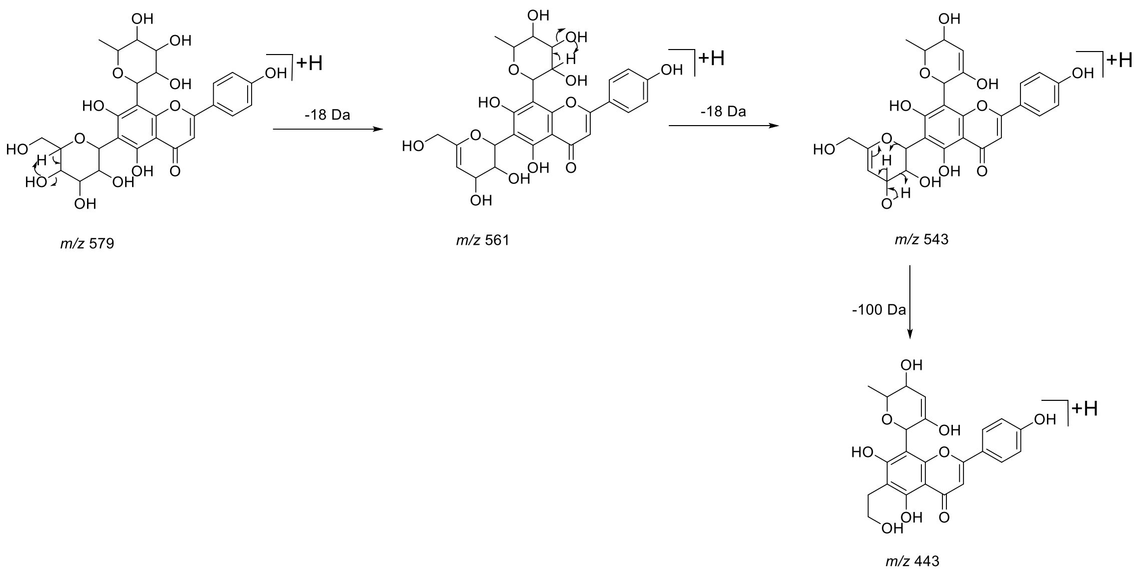

Scheme S19. Proposed fragmentation of apigenin 6-*C*-hexosyl-8-*C*-deoxihexoside in positive mode.

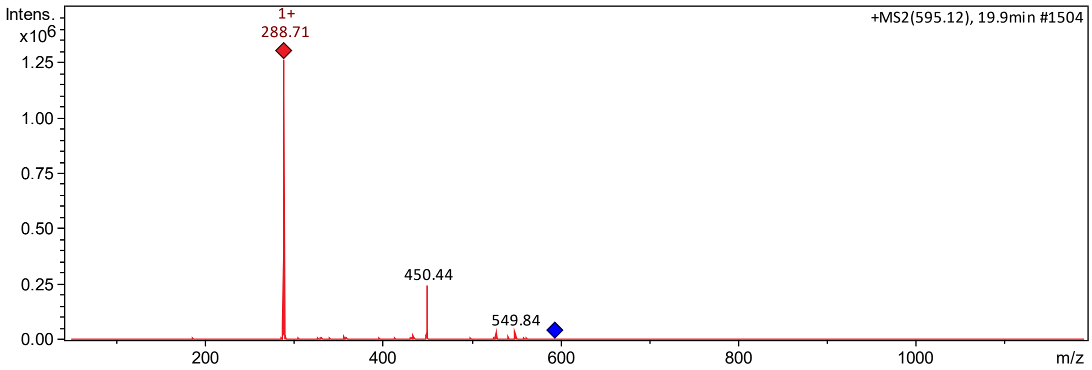

Figure S20. Mass spectrum of kaempferol *O*-deoxyhexosyl-*O*-hexoside.

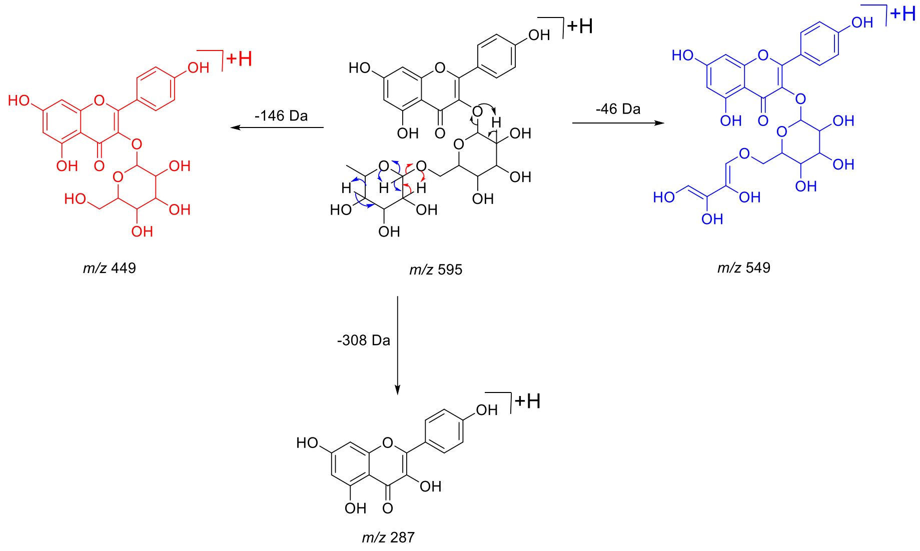

Scheme S20. Proposed fragmentation of apigenin kaempferol *O*-deoxyhexosyl-*O*-hexoside in positive mode.
